# Electrically switchable chiral nonlinear optics in an achiral ferroelectric 2D van der Waals halide perovskite

**DOI:** 10.1126/sciadv.adq5521

**Published:** 2024-11-13

**Authors:** Go Yumoto, Fuyuki Harata, Tomoya Nakamura, Atsushi Wakamiya, Yoshihiko Kanemitsu

**Affiliations:** Institute for Chemical Research, Kyoto University, Uji, Kyoto 611-0011, Japan.

## Abstract

Two-dimensional (2D) van der Waals (vdW) semiconductors play a key role in developing nanoscale nonlinear optical devices. 2D Ruddlesden-Popper lead halide perovskites (RPPs) expand the potential of using 2D vdW semiconductors in nonlinear optical applications because they exhibit electrically switchable and chiral second-order optical nonlinearity originating from the emergence of ferroelectricity and chirality. However, electrically switchable chiral nonlinear optics has not yet been realized because of the difficulty in electrically manipulating chiral structures. Here, we demonstrate that chiral second-harmonic generation (SHG) can be electrically induced and switched in an achiral biaxial ferroelectric 2D RPP. We observe reversible and continuous electrical switching of SHG circular dichroism and large nonlinear chiroptical activity. Polarization-resolved SHG imaging reveals that electrical poling induces the ferroelectric multidomain structure arising from the biaxial nature of the material, and the planar chirality appears. Our findings show a simple electrical control of the nonlinear chiroptical responses and establish chiral nonlinear optics based on ferroelectric 2D RPPs.

## Teaser

Ferroelectric 2D halide perovskites realize electrically switchable chiral SHG and boost the potential of chiral nonlinear optics.

## INTRODUCTION

Two-dimensional (2D) van der Waals (vdW) semiconductors have great potential for next-generation photonic and optoelectronic applications because of their properties such as strong light-matter interactions and compatibility with on-chip integration (*1*, *2*). The lack of inversion symmetry in non-centrosymmetric 2D vdW semiconductors provides another distinctive feature of efficient second-order nonlinear optical responses, such as second-harmonic generation (SHG) and the bulk photovoltaic effect (*3*–*7*). In addition, recent developments in 2D vdW ferroelectrics (*8*) have extended the nonlinear optical functionality of 2D vdW semiconductors for their strong and electrically switchable second-order optical nonlinearity (*9*, *10*).

2D Ruddlesden-Popper lead halide perovskites (RPPs), L_2_A_*n*−1_Pb*_n_*X_3*n*+1_, are an emerging class of 2D vdW semiconductors with excellent photonic (*11*, *12*), optoelectronic (*13*, *14*), and spintronic (*15*, *16*) properties. Here, L and A are bulky and smaller organic cations, respectively, X is a halide, and *n* is an integer. A 2D perovskite layer A_*n*−1_Pb*_n_*X_3*n*+1_ consisting of *n* layers of corner-sharing [PbX_6_]^4−^ octahedra is sandwiched between L spacer cations, and vdW interactions between the two L spacer cations assemble the unit layers into a multiple-quantum-well structure (*17*, *18*). 2D RPPs have high structural flexibility and tunability (*19*, *20*), and ferroelectricity emerges by introducing asymmetry or chirality into the organic spacer cations (*21*–*24*) or by using large A-site cations (*25*–*30*). Ferroelectric 2D RPPs have been synthesized with various compositions, and multiaxial ferroelectrics have been realized (*25*, *26*, *28*–*30*). Unlike uniaxial ferroelectrics, multiaxial ferroelectrics have multiple equivalent directions of spontaneous polarization and, hence, provide a unique way to electrically control the polarization direction (*31*). Another notable property of ferroelectric 2D RPPs is the simultaneous realization of chirality and ferroelectricity by using chiral organic spacer cations (*24*). In a chiral material, where its mirror image cannot be superimposed on itself, the optical responses depend on the handedness of circularly polarized light and chiroptical activity appears. Because chiroptical activity also manifests itself in nonlinear processes (*32*–*38*), 2D RPPs with coexistent chirality and ferroelectricity would provide a platform for electrically switchable chiral nonlinear optics. However, so far, this has not been realized in 2D vdW semiconductors because it is difficult to electrically manipulate the chiral structure of solid-state materials. Only a few experimental studies have reported electrical switching of chirality in solids and those relied on careful materials design (*34*, *39*). Among those studies, electrically switchable nonlinear chiroptical responses have been demonstrated only in oxide superlattices (*34*). Moreover, in contrast to the large family of 2D RPPs exhibiting either chirality or ferroelectricity (*40*, *41*), 2D RPPs having both properties are very scarce (*24*). Therefore, a versatile approach is desirable for developing electrically switchable chiral nonlinear optics and enriching the nonlinear optical functionalities of 2D vdW semiconductors.

Here, we demonstrated reversible and continuous electrical switching of SHG circular dichroism (SHG-CD), a second-order nonlinear chiroptical effect, in an achiral biaxial ferroelectric 2D RPP and observed a large SHG-CD. By performing polarization-resolved SHG imaging, we found that electrical poling induces and modulates the ferroelectric domains with perpendicular spontaneous polarizations, reflecting the biaxial nature of the material. The resulting ferroelectric multidomain structure breaks the in-plane glide mirror symmetry, and planar chirality emerges from achiral ferroelectric 2D RPP crystals. Our findings suggest an approach for simple electrical control of nonlinear chiroptical responses based on ferroelectric 2D RPPs.

## RESULTS

### Crystal symmetry and optical properties

**Fig. 1.**
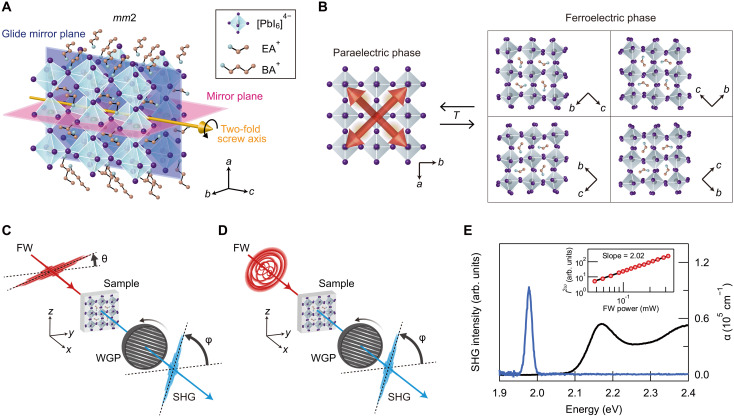
Crystal symmetry and optical responses in ferroelectric 2D RPP (BA)_2_(EA)_2_Pb_3_I_10_ crystals and optical setup of polarization-resolved SHG imaging. (**A**) Crystal structure of ferroelectric (BA)_2_(EA)_2_Pb_3_I_10_ with *mm*2 point group symmetry. (**B**) Top view of 2D perovskite layers in higher-temperature paraelectric (left) and lower-temperature ferroelectric (right) phases. The red arrows show the four equivalent [110] directions in the paraelectric phase. The schematic diagrams in the right boxes show four equivalent directions of the crystallographic *c* axis in the ferroelectric phase, which coincide with the equivalent [110] directions in the paraelectric phase. (**C** and **D**) Illustrations of optical setups for measuring SHG-RA (C) and SHG-CD (D) using linearly and circularly polarized FWs. θ and φ represent the rotation angles of the linearly polarized FW and the transmission axis of the analyzing WGP, respectively, which are measured from the *y* axis of the laboratory coordinate system. The FWs propagate along the *x* axis. (**E**) SHG (blue curve; left axis) and absorption coefficient (black curve; right axis) spectra of ferroelectric (BA)_2_(EA)_2_Pb_3_I_10_ at room temperature. The SHG spectrum was obtained at an excitation power of 3 × 10^−1^ mW. Inset: Excitation power dependence of SHG intensity. The black line is a linear fit to the data in the log-log plot with a slope of 2.02.

We synthesized 2D RPP (BA)_2_(EA)_2_Pb_3_I_10_ single crystals (Materials and Methods), which exhibit room-temperature biaxial ferroelectricity (*25*, *42*). Here, BA stands for butylammonium and EA for ethylammonium. X-ray diffraction patterns of the samples ensured the purity of the *n* = 3 phase, wherein all of the diffraction peaks could be assigned to (BA)_2_(EA)_2_Pb_3_I_10_ (fig. S1) (*43*). Figure 1A illustrates the crystal structure and symmetry of ferroelectric (BA)_2_(EA)_2_Pb_3_I_10_, where the crystallographic *a* axis is directed perpendicular to the 2D perovskite layer and the crystallographic *b* and *c* axes are oriented parallel to the 2D layer. In the ferroelectric phase, (BA)_2_(EA)_2_Pb_3_I_10_ crystals belong to the achiral space group *Cmc*2_1_ (point group *mm*2) (*25*, *43*, *44*) and contain a mirror plane parallel to the *bc* plane, a glide mirror plane parallel to the *ac* plane, and a twofold screw axis along the *c* axis (Fig. 1A). The spontaneous polarization of ferroelectric (BA)_2_(EA)_2_Pb_3_I_10_ is directed along the *c* axis. The biaxial ferroelectricity originates from four equivalent polarization directions coinciding with the equivalent [110] directions in the higher-temperature paraelectric phase (Fig. 1B) (*25*, *42*).

We performed polarization-resolved SHG imaging of exfoliated flakes of ferroelectric (BA)_2_(EA)_2_Pb_3_I_10_ by using linearly (Fig. 1C) and circularly (Fig. 1D) polarized fundamental waves (FWs) at normal incidence to measure SHG-RA and SHG-CD signals, respectively (Materials and Methods). For the SHG-RA measurements (Fig. 1C), we simultaneously rotated the linear polarization of the FW and the analyzing wire grid polarizer (WGP) by setting φ = θ (parallel configuration) or φ = θ + 90° (perpendicular configuration) and obtained the θ dependence of the SHG intensity, i.e., $I_{\text{para}}^{2\omega }\left(\theta \right)$ or $I_{\text{perp}}^{2\omega }\left(\theta \right)$. We also measured the φ dependence of the SHG intensities with right-handed (σ^+^) and left-handed (σ^−^) circularly polarized FWs, i.e., $I_{\sigma^{+}}^{2\omega }\left(\varphi \right)$ and $I_{\sigma^{-}}^{2\omega }\left(\varphi \right)$ (Fig. 1D). SHG-CD is defined as $\left(I_{\sigma^{+}}^{2\omega }-I_{\sigma^{-}}^{2\omega }\right)/\left(I_{\sigma^{+}}^{2\omega }+I_{\sigma^{-}}^{2\omega }\right)$, where $I_{\sigma^{+}}^{2\omega }$ ($I_{\sigma^{-}}^{2\omega }$) denotes the total SHG intensity with σ^+^ (σ^−^) circularly polarized FW (*40*, *45*) (Materials and Methods). It is known that SHG-CD is a good probe of chiroptical activity with much higher sensitivity compared with linear circular dichroism (*32*, *35*, *36*). Note that the crystallographic *bc* plane of the samples was parallel to the *yz* plane of the laboratory coordinate system in our experiments.

Figure 1E shows the SHG and absorption coefficient spectra of ferroelectric (BA)_2_(EA)_2_Pb_3_I_10_ at room temperature. The absorption coefficient spectrum was obtained by measuring the reflectance and transmittance of an exfoliated sample whose thickness was estimated by atomic force microscopy (Supplementary Text S1) (*16*). The SHG spectrum centered at 1.98 eV (627 nm) was obtained using an FW with a photon energy of 0.99 eV (1253 nm). The inset shows the SHG intensity as a function of FW power, exhibiting a quadratic power dependence, as expected from the SHG process. In the experiments described below, we fixed the FW photon energy and power to be 0.99 eV and 3 × 10^−1^ mW to prevent multiphoton absorption of the FW and direct absorption of SHG.

### Polarization-resolved SHG imaging of as-prepared samples

**Fig. 2.**
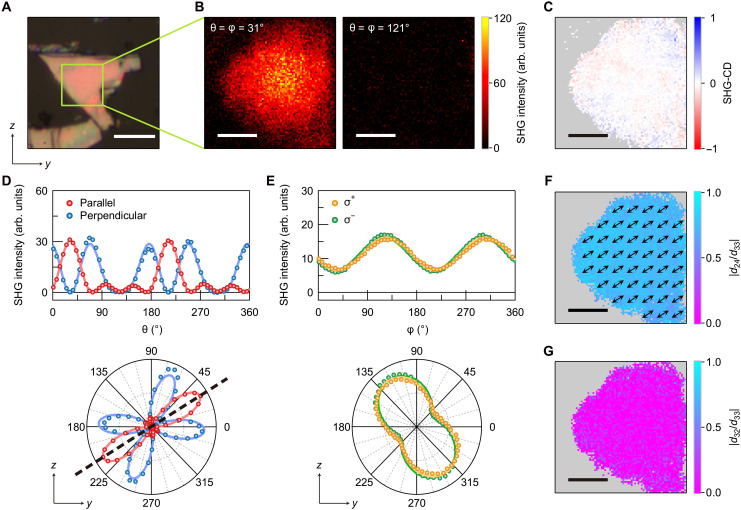
SHG responses from an as-prepared (BA)_2_(EA)_2_Pb_3_I_10_ flake. (**A**) Optical microscope image of an exfoliated sample with the *y* and *z* axes of the laboratory coordinate system. Scale bar, 10 μm. (**B**) Maps of the SHG intensity generated by the linearly polarized FW with θ = φ = 31° (left) and θ = φ = 121° (right). The displayed region corresponds to the area marked by the green square in (A). Scale bars, 3 μm. (**C**) Map of SHG-CD obtained in the same spatial region as shown in (B). Scale bar, 3 μm. (**D**) Top: θ dependence of the SHG intensities obtained in the parallel (red circles) and perpendicular (blue circles) configurations of the SHG-RA measurements. The curves are fits using Eq. 1. Bottom: Polar plots of the SHG intensities shown in the top panel. The black dashed line is along the crystallographic *c* axis and shows the orientation of the glide mirror plane. (**E**) Top: φ dependence of the SHG intensities with σ^+^ (orange circles) and σ^−^ (green circles) circularly polarized FWs. The curves are fits using Eq. 2. Bottom: Polar plots of the SHG intensities shown in the top panel. The SHG intensities in (D) and (E) are averages of the signals over the whole region shown in (B). The corresponding *y* and *z* axes are also depicted in the polar plots in (D) and (E). (**F** and **G**) Maps of ∣*d*_24_∣/∣*d*_33_∣ (F) and ∣*d*_32_∣/∣*d*_33_∣ (G) estimated in the same spatial region as shown in (B). The black double-sided arrows in (F) indicate the two possible directions of the *c* axis at each spatial position. Scale bars, 3 μm. The gray-shaded areas in (C), (F), and (G) correspond to the regions where the related SHG intensities are weak or not observable.

Figure 2A shows an optical microscope image of an as-prepared (BA)_2_(EA)_2_Pb_3_I_10_ flake with a thickness of 340 nm on a glass substrate. Figure 2B shows spatial maps of the SHG intensities in the parallel configuration of the SHG-RA measurements obtained from the area of this sample marked by the green square in Fig. 2A. The SHG intensity maps were taken by irradiating the homogeneous region of the sample with the FW having a beam spot size of ~8 μm at full width at half maximum at the sample position (fig. S3). In contrast to the clear SHG signal for θ = φ = 31°, no SHG signal was observed for θ = φ = 121°, thereby indicating a strongly anisotropic SHG response. Figure 2C illustrates the SHG-CD map; zero SHG-CD was observed over the whole spatial region, and it confirms the absence of chiroptical activity as expected from the in-plane glide mirror symmetry of the sample.

To further characterize the as-prepared sample, we plotted $I_{\text{para}}^{2\omega }\left(\theta \right)$ and $I_{\text{perp}}^{2\omega }\left(\theta \right)$ (Fig. 2D) and $I_{\sigma^{+}}^{2\omega }\left(\varphi \right)$ and $I_{\sigma^{-}}^{2\omega }\left(\varphi \right)$ (Fig. 2E). The plotted $I_{\text{para}}^{2\omega }\left(\theta \right)$ and $I_{\text{perp}}^{2\omega }\left(\theta \right)$ show substantial θ dependences, reflecting the strong SHG anisotropy, while $I_{\sigma^{+}}^{2\omega }\left(\varphi \right)$ and $I_{\sigma^{-}}^{2\omega }\left(\varphi \right)$ exhibit similar sinusoidal φ dependences. The good agreement between $I_{\sigma^{+}}^{2\omega }\left(\varphi \right)$ and $I_{\sigma^{-}}^{2\omega }\left(\varphi \right)$ is consistent with the zero SHG-CD shown in Fig. 2C. Because of the *mm*2 point group symmetry, the SHG signals in our experimental configurations can be described using three independent tensor elements, $d_{24}=\frac{1}{2}\chi_{\mathrm{yyz}}^{\left(2\right)}=\frac{1}{2}\chi_{\mathrm{yzy}}^{\left(2\right)}$, $d_{32}=\frac{1}{2}\chi_{\mathrm{zyy}}^{\left(2\right)}$, and $d_{33}=\frac{1}{2}\chi_{\mathrm{zzz}}^{\left(2\right)}$, where $\chi_{\mathrm{ijk}}^{\left(2\right)}$ is the second-order nonlinear optical susceptibility (Materials and Methods) (*46*, *47*). By calculating the polarization dependence of the SHG intensity (Materials and Methods), we found that $I_{\text{para}}^{2\omega }\left(\theta \right)$ and $I_{\text{perp}}^{2\omega }\left(\theta \right)$ can be expressed as

$$A_{\text{lin}}+B_{\text{lin}}\text{cos}\left[2\left(\theta -\theta_{b}\right)\right]+C_{\text{lin}}\text{cos}\left[4\left(\theta -\theta_{b}\right)\right]+D_{\text{lin}}\text{cos}\left[6\left(\theta -\theta_{b}\right)\right]$$ (1)

where θ*_b_* is the orientation angle of the *b* axis relative to the *y* axis and the coefficients of $I_{\text{para}}^{2\omega }\left(\theta \right)$ are different from those of $I_{\text{perp}}^{2\omega }\left(\theta \right)$. In addition, $I_{\sigma^{+}}^{2\omega }\left(\varphi \right)$ and $I_{\sigma^{-}}^{2\omega }\left(\varphi \right)$ can be described by

$$E_{\text{circ}}+F_{\text{circ}}\text{cos}\left[2\left(\varphi -\theta_{b}\right)\right]\pm G_{\text{circ}}\text{sin}\left[2\left(\varphi -\theta_{b}\right)\right]$$ (2)

where + (−) sign is for $I_{\sigma^{+}}^{2\omega }\left(\varphi \right)$ [$I_{\sigma^{-}}^{2\omega }\left(\varphi \right)$]. The solid curves in Fig. 2 (D and E) are fits using Eqs. 1 and 2, respectively, and their good agreement with experimental data corroborates the *mm*2 point group symmetry of the sample. From the fits to $I_{\text{para}}^{2\omega }\left(\theta \right)$ and $I_{\text{perp}}^{2\omega }\left(\theta \right)$, we determined the orientation angle of the *c* axis relative to the *y* axis, θ*_c_*, to be 32°. The polar plot in Fig. 2D clearly shows that the SHG-RA patterns are symmetric with respect to the *c* axis, which reflects the presence of a glide mirror plane parallel to the *ac* plane.

The fitting also gives the ratios between the nonlinear optical tensor elements of ∣*d*_24_∣/∣*d*_33_∣ and ∣*d*_32_∣/∣*d*_33_∣ through the following relations: $I_{\text{perp}}^{2\omega }\left(\theta_{b}\right)\propto {|d_{32}|}^{2}$, $I_{\text{para}}^{2\omega }\left(\theta_{c}\right)\propto {|d_{33}|}^{2}$, and $I_{\sigma^{+}}^{2\omega }\left(\theta_{b}\right)=I_{\sigma^{-}}^{2\omega }\left(\theta_{b}\right)\propto {|d_{24}|}^{2}$ (Materials and Methods). Figure 2 (F and G) shows the estimated spatial maps of ∣*d*_24_∣/∣*d*_33_∣ and ∣*d*_32_∣/∣*d*_33_∣. Figure 2F also shows the orientation of the *c* axis estimated at each spatial position. We found that these quantities are homogeneously distributed across the sample and estimated the spatially averaged values to be ∣*d*_24_∣/∣*d*_33_∣ = 0.74 ± 0.05, ∣*d*_32_∣/∣*d*_33_∣ = 0.1 ± 0.1, and θ*_c_* = 32° ± 2°. The small value of ∣*d*_32_∣/∣*d*_33_∣ shows that ∣*d*_32_∣ is negligible compared to ∣*d*_24_∣ and ∣*d*_33_∣. We confirmed that the values of ∣*d*_24_∣/∣*d*_33_∣ and ∣*d*_32_∣/∣*d*_33_∣ did not change when we measured different crystal flakes (fig. S4).

### Electrically switchable chiral SHG

**Fig. 3.**
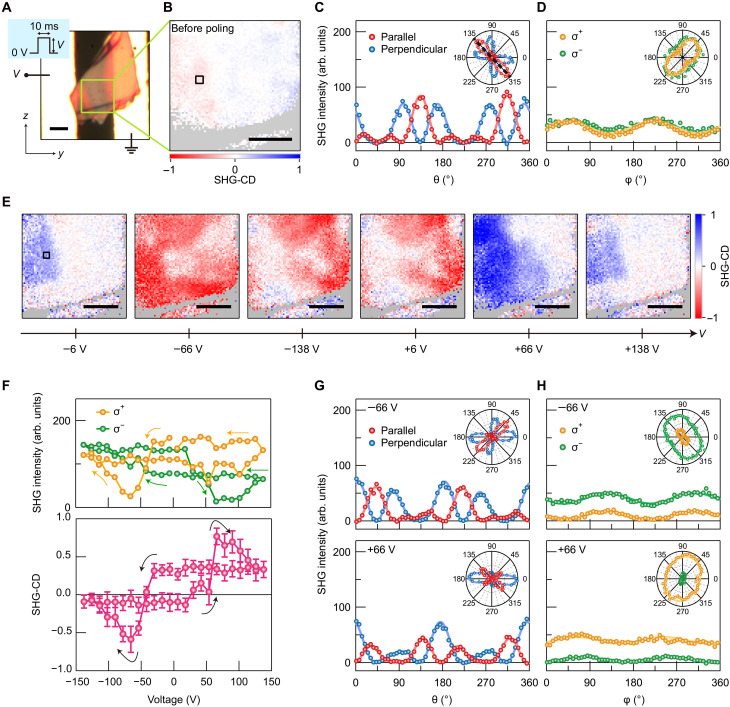
Electrically induced and controlled nonlinear chiroptical responses. (**A**) Optical microscope image of an exfoliated sample on interdigitated electrodes. The inset shows a schematic diagram of the voltage pulse. Scale bar, 5 μm. (**B**) Map of the SHG-CD before applying the voltage pulses. The displayed region corresponds to the area marked by the green square in (A). Scale bar, 3 μm. (**C**) θ dependence of the SHG intensities before poling obtained in the parallel (red circles) and perpendicular (blue circles) configurations of the SHG-RA measurements. (**D**) φ dependence of the SHG intensities before poling with σ^+^ (orange circles) and σ^−^ (green circles) circularly polarized FWs. The curves in (C) and (D) are fits using Eqs. 1 and 2, respectively. The insets show the corresponding polar plots. The black dashed line in the inset of (C) is along the crystallographic *c* axis. (**E**) A series of maps of the SHG-CD acquired during the poling process. The boxed region is the same as the one in (B). Scale bars, 3 μm. (**F**) Hysteresis of the total SHG intensities with σ^+^ and σ^−^ (top; orange and green circles, respectively) circularly polarized FWs and the corresponding SHG-CD (bottom; pink circles). The arrows indicate the switching sequence. The error bars show the standard deviation of spatially averaged values. (**G** and **H**) Same as (C) and (D) but after applying voltage pulses of −66 V (top) and +66 V (bottom). The curves in (G) and (H) are fits using Eqs. 3 and 4, respectively. The SHG signals shown in (C) and (D) and (F) to (H) are averages of the signals in the area marked by the black square in (B) and (E). The gray-shaded areas in (B) and (E) correspond to the regions where the related SHG intensities are weak or not observable.

To demonstrate electrical switching of the SHG signals, an exfoliated flake with a thickness of 540 nm was transferred onto gold interdigitated electrodes with a spacing of 10 μm (Fig. 3A). Figure 3A also shows the *y* and *z* axes of the laboratory coordinate system, and the bright regions in Fig. 3A correspond to the gold electrodes, with the right electrode connected to ground. The sample was electrically poled by sequentially changing the amplitude of the applied voltage pulses with a duration of 10 ms (see the inset of Fig. 3A and Materials and Methods). Figure 3B displays the spatial map of the SHG-CD measured before poling, which shows zero SHG-CD over the whole region. As in the case of the as-prepared samples, $I_{\text{para}}^{2\omega }\left(\theta \right)$ and $I_{\text{perp}}^{2\omega }\left(\theta \right)$ (Fig. 3C) and $I_{\sigma^{+}}^{2\omega }\left(\varphi \right)$ and $I_{\sigma^{-}}^{2\omega }\left(\varphi \right)$ (Fig. 3D) were well reproduced under the assumption of *mm*2 point group symmetry, and we observed homogeneous spatial distributions of ∣*d*_24_∣/∣*d*_33_∣ (=0.67 ± 0.05), ∣*d*_32_∣/∣*d*_33_∣ (=0.09 ± 0.09), and θ*_c_* (=133.2° ± 0.9°) (fig. S5). The *c* axis was tilted by 41° from the direction parallel to the electrodes.

Figure 3E illustrates a series of the SHG-CD maps that were acquired by applying sequential voltage pulses to the sample. The amplitude of the voltage pulse *V* was sequentially changed as follows: *V* started at −6 V, decreased to −138 V, increased to +138 V, and decreased to +6 V in increments of 12 V. Unexpectedly, nonlinear chiroptical responses were electrically induced and switched in an achiral (BA)_2_(EA)_2_Pb_3_I_10_ flake. A positive SHG-CD signal appeared at *V* = −6 V, and the sign of the signal reversed with decreasing *V* and stayed negative up to *V* = +6 V. Upon increasing *V* further, the sign became positive again. We confirmed that the switching behavior could be reproduced in different voltage cycles (fig. S6) and that it appeared in different crystal flakes (Supplementary Text S2). As shown in Fig. 3F, we can see a hysteretic nature in the voltage dependences of $I_{\sigma^{+}}^{2\omega }$, $I_{\sigma^{-}}^{2\omega }$, and SHG-CD, which were averaged within the region in Fig. 3E enclosed by the black line. When *V* is decreased to −66 V, only $I_{\sigma^{+}}^{2\omega }$ is suppressed and the SHG-CD signal takes a minimum of −0.6. When *V* is increased to +66 V, the SHG-CD signal reaches a maximum of +0.8 accompanying the suppression of $I_{\sigma^{-}}^{2\omega }$. The observed maximum SHG-CD is larger than the values reported for chiral halide perovskites (*33*, *35*, *36*, *48*) and is close to those reported in plasmonic metasurfaces that show large nonlinear chiroptical activity (*49*).

To gain deeper insight into the SHG-CD signals, we plotted $I_{\text{para}}^{2\omega }\left(\theta \right)$ and $I_{\text{perp}}^{2\omega }\left(\theta \right)$ [$I_{\sigma^{+}}^{2\omega }\left(\varphi \right)$ and $I_{\sigma^{-}}^{2\omega }\left(\varphi \right)$] for *V* = −66 and +66 V [Fig. 3G (Fig. 3H)]. The voltages shown in Fig. 3 (G and H) correspond to those where the SHG-CD hysteresis in Fig. 3F takes a minimum and a maximum value. Note that the signals in Fig. 3 (G and H) were obtained in the same spatial region as the signals in Fig. 3 (C and D), which corresponds to the area marked by the black square in Fig. 3 (B and E). We found that the SHG-RA patterns markedly changed depending on the applied voltage and had no symmetric axis. This contrasts with the SHG-RA pattern before poling, which was symmetric with respect to the *c* axis (see Fig. 3C). In addition, Fig. 3H shows substantial differences between $I_{\sigma^{+}}^{2\omega }\left(\varphi \right)$ and $I_{\sigma^{-}}^{2\omega }\left(\varphi \right)$ at every φ, i.e., SHG-CD. None of these signals for *V* = −66 and +66 V could be accounted for under *mm*2 point group symmetry. The absence of a symmetric axis in the SHG-RA patterns and the appearance of the SHG-CD indicate the breaking of the glide mirror symmetry as a result of applying the voltage pulses. Therefore, we must consider a lower symmetry subgroup of point group *mm*2 for describing the observed chiral SHG.

Among the lower symmetry subgroups, only point group 1 with no mirror and rotational symmetries or point group *m* with one mirror plane parallel to the *yz* plane and no rotational symmetry could reproduce our results; both groups have no in-plane mirror symmetry (Materials and Methods). Note that point group 1 is a chiral point group, while point group *m* is achiral. The SHG responses for these point group symmetries can be determined by six independent tensor elements: $d_{22}=\frac{1}{2}\chi_{\mathrm{yyy}}^{\left(2\right)}$, $d_{23}=\frac{1}{2}\chi_{\mathrm{yzz}}^{\left(2\right)}$, $d_{24}=\frac{1}{2}\chi_{\mathrm{yyz}}^{\left(2\right)}=\frac{1}{2}\chi_{\mathrm{yzy}}^{\left(2\right)}$, $d_{32}=\frac{1}{2}\chi_{\mathrm{zyy}}^{\left(2\right)}$, $d_{33}=\frac{1}{2}\chi_{\mathrm{zzz}}^{\left(2\right)}$, and $d_{34}=\frac{1}{2}\chi_{\mathrm{zyz}}^{\left(2\right)}=\frac{1}{2}\chi_{\mathrm{zzy}}^{\left(2\right)}$. We found that $I_{\text{para}}^{2\omega }\left(\theta \right)$ and $I_{\text{perp}}^{2\omega }\left(\theta \right)$ can be expressed in the form

$$\begin{array}{c} A_{\text{lin}}^{\prime }+B_{\text{lin}}^{\prime }\text{cos}\left[2\left(\theta -\theta_{b}^{\prime }\right)\right]+C_{\text{lin}}^{\prime }\text{cos}\left[4\left(\theta -\theta_{b}^{\prime }\right)\right]+D_{\text{lin}}^{\prime }\text{cos}\left[6\left(\theta -\theta_{b}^{\prime }\right)\right]\\ +E_{\text{lin}}^{\prime }\text{sin}\left[2\left(\theta -\theta_{b}^{\prime }\right)\right]+F_{\text{lin}}^{\prime }\text{sin}\left[4\left(\theta -\theta_{b}^{\prime }\right)\right]+G_{\text{lin}}^{\prime }\text{sin}\left[6\left(\theta -\theta_{b}^{\prime }\right)\right] \end{array}$$ (3)

and $I_{\sigma^{+}}^{2\omega }\left(\varphi \right)$ and $I_{\sigma^{-}}^{2\omega }\left(\varphi \right)$ in the form

$$H_{\text{circ}}^{\prime }+I_{\text{circ}}^{\prime }\text{cos}\left[2\left(\varphi -\theta_{b}^{\prime }\right)\right]+J_{\text{circ}}^{\prime }\text{sin}\left[2\left(\varphi -\theta_{b}^{\prime }\right)\right]$$ (4)

where the coefficients for $I_{\text{para}}^{2\omega }\left(\theta \right)$ [$I_{\sigma^{+}}^{2\omega }\left(\varphi \right)$] are different for those of $I_{\text{perp}}^{2\omega }\left(\theta \right)$ [$I_{\sigma^{-}}^{2\omega }\left(\varphi \right)$]. The experimental results in Fig. 3 (G and H) are well reproduced by Eqs. 3 and 4, respectively, where we set $\theta_{b}^{\prime }$ = 43° corresponding to θ*_b_* before poling. This shows that the chiral SHG originates from the emergence of the planar chirality due to the electrically induced breaking of the in-plane glide mirror symmetry.

**Fig. 4.**
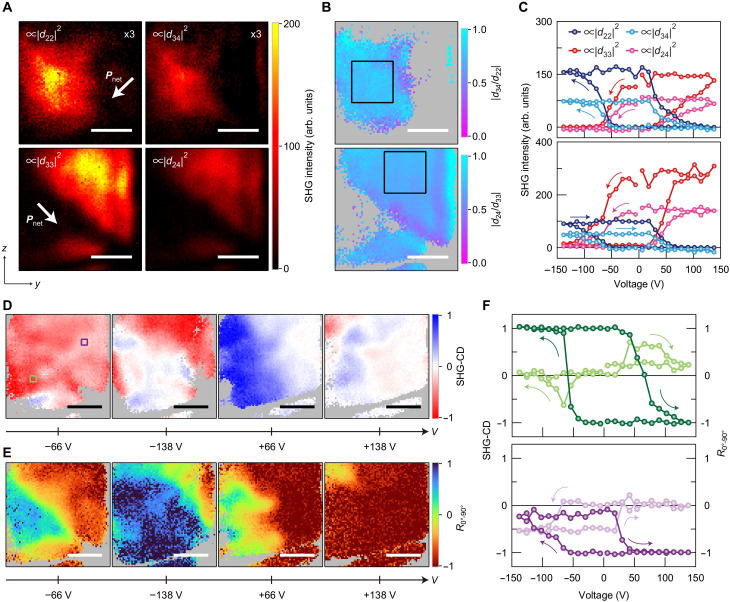
Origin of the electrically switchable chiral SHG. (**A**) Maps of SHG intensities proportional to ∣*d*_22_∣^2^ (top left), ∣*d*_34_∣^2^ (top right), ∣*d*_33_∣^2^ (bottom left), and ∣*d*_24_∣^2^ (bottom right) acquired after applying voltage pulses of −66 V. The SHG intensities of ∣*d*_22_∣^2^ and ∣*d*_34_∣^2^ are magnified by a factor of 3. The white arrow in the top (bottom) left panel depicts the direction of the net spontaneous polarization of the domains characterized by ∣*d*_22_∣^2^ and ∣*d*_34_∣^2^ (∣*d*_33_∣^2^ and ∣*d*_24_∣^2^). Scale bars, 3 μm. (**B**) Maps of the estimated ∣*d*_34_∣/∣*d*_22_∣ (top) and ∣*d*_24_∣/∣*d*_33_∣ (bottom). Scale bars, 3 μm. (**C**) Hysteresis of SHG intensities proportional to ∣*d*_22_∣^2^ (dark blue circles), ∣*d*_34_∣^2^ (light blue circles), ∣*d*_33_∣^2^ (dark red circles), and ∣*d*_24_∣^2^ (light red circles). The top (bottom) panel shows the SHG intensities obtained by averaging the signals in the area marked by the black square in the top (bottom) panel of (B). (**D** and **E**) A series of maps of SHG-CD (D) and degree of the spontaneous polarization state (E) obtained during the poling process. Scale bars, 3 μm. (**F**) Top: Hysteresis of SHG-CD (light green circles; left axis) and degree of the spontaneous polarization state (dark green circles; right axis) obtained by averaging the signals in the area marked by the green square in the leftmost panel of (D). Bottom: Same as the top panel, but for the area marked by the purple square in the leftmost panel of (D). The arrows in the panels of (C) and (F) indicate the switching sequence. The region displayed in (A), (B), (D), and (E) corresponds to the one in Fig. 3B. The gray-shaded areas in (B), (D), and (E) correspond to the regions where the related SHG intensities are weak or not observable.

To clarify the symmetry-breaking mechanism, we determined the SHG intensities directly related to each of the six tensor elements by using the following relations: $I_{\text{para}}^{2\omega }\left(\theta_{b}^{\prime }\right)\propto {|d_{22}|}^{2}$, $I_{\text{perp}}^{2\omega }\left(\theta_{b}^{\prime }\right)\propto {|d_{32}|}^{2}$, $I_{\text{para}}^{2\omega }\left(\theta_{c}^{\prime }\right)\propto {|d_{33}|}^{2}$, $I_{\text{perp}}^{2\omega }\left(\theta_{c}^{\prime }\right)\propto {|d_{23}|}^{2}$, $\frac{I_{\sigma^{+}}^{2\omega }\left(\theta_{b}^{\prime }\right)+I_{\sigma^{-}}^{2\omega }\left(\theta_{b}^{\prime }\right)}{2}-\frac{I_{\text{para}}^{2\omega }\left(\theta_{b}^{\prime }\right)}{4}\propto {|d_{24}|}^{2}$, and $\frac{I_{\sigma^{+}}^{2\omega }\left(\theta_{c}^{\prime }\right)+I_{\sigma^{-}}^{2\omega }\left(\theta_{c}^{\prime }\right)}{2}-\frac{I_{\text{para}}^{2\omega }\left(\theta_{c}^{\prime }\right)}{4}\propto {|d_{34}|}^{2}$, where $\theta_{c}^{\prime }=\theta_{b}^{\prime }+90{}^{\circ}$ (Materials and Methods). Figure 4A illustrates the spatial maps of the estimated SHG intensities proportional to ∣*d*_22_∣^2^, ∣*d*_34_∣^2^, ∣*d*_33_∣^2^, and ∣*d*_24_∣^2^ for *V* = −66 V, where the SHG-CD in Fig. 3F takes a minimum value. The SHG intensity maps proportional to ∣*d*_23_∣^2^ and ∣*d*_32_∣^2^ are not displayed because these SHG signals were too weak to be observed at any of the applied voltages. We found two different patterns in the spatial distribution of the SHG intensities, i.e., one for ∣*d*_22_∣^2^ and ∣*d*_34_∣^2^ and the other for ∣*d*_33_∣^2^ and ∣*d*_24_∣^2^. The similarity in the spatial patterns of ∣*d*_22_∣^2^ and ∣*d*_34_∣^2^ (∣*d*_33_∣^2^ and ∣*d*_24_∣^2^) can also be seen in the homogeneous spatial distribution of ∣*d*_34_∣/∣*d*_22_∣ (∣*d*_24_∣/∣*d*_33_∣) shown in Fig. 4B. By averaging the values within the regions in Fig. 4B enclosed by the black squares, we obtained ∣*d*_34_∣/∣*d*_22_∣ = 0.75 ± 0.06 and ∣*d*_24_∣/∣*d*_33_∣ = 0.69 ± 0.04. These values agree well with that of ∣*d*_24_∣/∣*d*_33_∣ before poling. Figure 4C shows the voltage dependence of the element-resolved SHG intensities averaged within the two different spatial regions. In both regions, the SHG hysteresis loops can be classified into two different patterns, i.e., one for ∣*d*_22_∣^2^ and ∣*d*_34_∣^2^ and the other for ∣*d*_33_∣^2^ and ∣*d*_24_∣^2^, keeping ∣*d*_34_∣/∣*d*_22_∣ and ∣*d*_24_∣/∣*d*_33_∣ constant (fig. S8).

The observed spatial and hysteresis patterns indicate the presence of two different types of domains: one characterized by tensor elements *d*_22_ and *d*_34_ and the other by *d*_33_ and *d*_24_. This is considered to originate from the coexistence of the domain variants reflecting biaxial ferroelectricity (see Fig. 1B). We found that the multidomain structure provides the same polarization dependences of SHG intensity as would be expected from 1 or *m* point group symmetries (Materials and Methods). Note that, because each domain variant has a mirror plane parallel to the *yz* plane due to *mm*2 point group symmetry, the multidomain structure is described by *m* point group symmetry. The model reveals that the tensor elements *d*_33_, *d*_24_, and *d*_32_ (*d*_22_, *d*_34_, and *d*_23_) for the low point group symmetries describes the SHG responses from the domain variants with the spontaneous polarizations directed at angles of 133° and 313° (223° and 43°) relative to the *y* axis, denoted as domains 1 and 2 (domains 3 and 4), respectively. The SHG intensities proportional to ∣*d*_33_∣^2^, ∣*d*_24_∣^2^, ∣*d*_22_∣^2^, and ∣*d*_34_∣^2^ for the low point group symmetries can be expressed as ∣δ*A*_0°_∣^2^∣*d*_33_∣^2^, ∣δ*A*_0°_∣^2^∣*d*_24_∣^2^, ∣δ*A*_90°_∣^2^∣*d*_33_∣^2^, and ∣δ*A*_90°_∣^2^∣*d*_24_∣^2^, respectively, using the tensor elements of each domain variant, δ*A*_0°_ = *A*_1_ − *A*_2_ and δ*A*_90°_ = *A*_3_ − *A*_4_ (Materials and Methods). Here, *A_i_* denotes the area fraction of domain *i* (*i* = 1 to 4) with *A*_1_ + *A*_2_ + *A*_3_ + *A*_4_ = 1. Because the spontaneous polarization directions of domains 1 and 2 (3 and 4) are antiparallel to each other, ∣δ*A*_0°_∣ (∣δ*A*_90°_∣) reflects the magnitude of the net spontaneous polarization. Note that, from the SHG hysteresis loops in Fig. 4C, we can determine the directions of the net spontaneous polarizations as indicated by the white arrows in Fig. 4A (Supplementary Text S3).

To investigate the correlation between the SHG-CD and the multidomain structure, we defined the degree of the spontaneous polarization state as *R*_0°-90°_ = (∣δ*A*_90°_∣ − ∣δ*A*_0°_∣)/(∣δ*A*_90°_∣ + ∣δ*A*_0°_∣). *R*_0°-90°_ was estimated from the SHG intensities proportional to ∣*d*_22_∣^2^ (∣δ*A*_90°_∣^2^∣*d*_33_∣^2^) and ∣*d*_33_∣^2^ (∣δ*A*_0°_∣^2^∣*d*_33_∣^2^). Here, −1 < *R*_0°-90°_ < 1 corresponds to the case where the domains with perpendicular spontaneous polarizations coexist below the SHG imaging resolution of ~900 nm, while *R*_0°-90°_ = +(−)1 shows that only the domain related to δ*A*_90°_ (δ*A*_0°_) has a net spontaneous polarization. Figure 4 (D and E) shows a series of spatial maps of SHG-CD and *R*_0°-90°_ that were acquired by applying sequential voltage pulses. The maps reveal that the spatial regions with SHG-CD ~ 0 show *R*_0°-90°_ ~ ±1, whereas the regions with finite SHG-CD signals exhibit −1 < *R*_0°-90°_ < 1. This correlation between SHG-CD and *R*_0°-90°_ is further confirmed by their hysteresis loops in Fig. 4F. Moreover, we found that, when SHG-CD ~ 0 and *R*_0°-90° _~ ±1, the SHG-RA patterns recover *mm*2 point group symmetry with different orientations of the *c* axis for *R*_0°-90°_ = +1 and −1 (Supplementary Text S4). These results verify our interpretation that the coexistence of the domains with perpendicular spontaneous polarizations breaks the in-plane glide mirror symmetry and the nonlinear chiroptical responses appear reflecting the multidomain structure below the optical resolution. In addition, it is found that the multidomain structure can be electrically induced and modulated when the spontaneous polarization before poling is tilted from the directions parallel or perpendicular to the electrodes (Fig. 3 and fig. S7). This indicates that the components of the applied electric field parallel to the two perpendicular spontaneous polarizations in the electrically induced multidomain structure are required to electrically switch the nonlinear chiroptical responses. These conclusions are further supported by the fact that SHG-CD was not electrically induced in the sample whose spontaneous polarization before poling was aligned perpendicular to the electrodes, where the poling cannot modulate the spontaneous polarization parallel to the electrodes (Supplementary Text S5). Note that the sign of SHG-CD is not determined by *R*_0°-90°_. From the expression of SHG-CD based on the ferroelectric multidomain structure, the sign change of SHG-CD is considered to originate from the electrical modulation of the relative phases between the second-order nonlinear polarizations from the domains with perpendicular spontaneous polarization directions (Materials and Methods).

The inhomogeneous spatial distribution of SHG-CD shown in Figs. 3E and 4D results from the fact that the hysteresis patterns of the two perpendicular net spontaneous polarizations related to ∣*d*_22_∣^2^ and ∣*d*_33_∣^2^ (Fig. 4A) are different depending on the spatial positions. This can be seen from the spatially dependent hysteresis loops of the SHG signals (Fig. 4C and fig. S11). The hysteresis loops of the SHG intensities shown in the bottom panel of Fig. 4C are shifted in the negative voltage direction compared to those shown in the top panel of Fig. 4C. Because the internal bias field based on defects and charges and the asymmetry of the electrodes are known to shift the ferroelectric polarization-electric field hysteresis (*50*), the spatial inhomogeneity of these effects would cause the observed inhomogeneous SHG hysteresis patterns. Although the inhomogeneity can be considered to reflect the inhomogeneous distributions of defects and charges, further studies are needed to clarify the microscopic origin of the inhomogeneity.

## DISCUSSION

We have demonstrated that nonlinear chiroptical responses can be electrically induced and switched in an achiral ferroelectric 2D RPP. The SHG-CD was electrically controlled in a reversible and continuous manner, and large SHG-CD was observed. The electrical switching of large nonlinear chiroptical activity would expand the potential of nonlinear optical applications such as frequency conversion, optical amplification, and photocurrent generation. In addition, such large nonlinear chiroptical responses show an advantage of using SHG-CD as a probe of chiroptical activity over using linear circular dichroism, which tends to be blurred by the signals related to the anisotropy of materials for linearly polarized light due to the weak linear circular dichroism signal (*51*). The polarization-resolved SHG imaging clarified that the electrically induced multidomain structure reflecting the biaxial ferroelectricity of the material breaks the in-plane glide mirror symmetry and causes the chiral SHG. By adjusting the angle between the directions of the spontaneous polarization before poling and the applied electric field, a systematic control of the chiral SHG can be expected. Our scheme for realizing and switching chiroptical activity is solely based on the biaxial nature of the ferroelectrics and does not require careful design and modulation of the chiral structure. Therefore, it widens the choice of materials for chiral nonlinear optics and provides an opportunity for simple electrical control of nonlinear chiroptical responses. These findings open avenues for chiral photonic and optoelectronic applications based on ferroelectric 2D RPPs.

## MATERIALS AND METHODS

### Sample preparation

We synthesized the (BA)_2_(EA)_2_Pb_3_I_10_ single crystals by following the previously reported procedure (*43*, *44*). Briefly, we dissolved PbI_2_ (690 mg, 1.5 mmol), EAI (173 mg, 1.0 mmol), and BAI (88 mg, 0.44 mmol) in a mixture of 57 wt % aqueous HI solution (2.2 ml, 16.8 mmol) and 50 wt % aqueous H_3_PO_2_ solution (0.28 ml, 2.5 mmol) by heating it to 120°C under constant magnetic stirring for about 5 min, yielding a bright-yellow solution. The solution was concentrated on a hot plate until the volume was reduced to two-thirds of the original volume. After that, red rectangular-shaped plates crystallized as the solution was cooled to 70°C at a rate of 5°C/hour. For characterizing the nonlinear optical properties of the as-prepared flakes, the (BA)_2_(EA)_2_Pb_3_I_10_ flakes were mechanically exfoliated and transferred onto a glass substrate. For the electrical switching measurements, the exfoliated flakes were transferred onto gold interdigitated electrodes with a spacing of 10 μm.

### Polarization-resolved SHG imaging

We used laser pulses with a wavelength of 1253 nm as the FWs, which were generated from an optical parametric amplifier (Orpheus, Light Conversion) pumped by a Yb:KGW regenerative amplifier (Pharos, Light Conversion) producing laser pulses with a central wavelength of 1028 nm with a repetition rate of 500 kHz. The 1253-nm beam passed through a Berek compensator and an achromatic quarter-wave plate, becoming circularly polarized. The circularly polarized beam was used as a FW for the SHG-CD measurements. For SHG-RA measurements, we used a WGP to obtain a linearly polarized FW from the circularly polarized beam. By rotating the WGP, we obtained the linear polarization of the FW rotated by θ relative to the *y* axis of the laboratory coordinate system (see Fig. 1C). In both the SHG-CD and SHG-RA measurements, the FW propagated along the *x* axis and was focused onto the sample from the substrate side by an objective lens [5×, numerical aperture (NA) = 0.14] at normal incidence. The beam spot size of the FW at the sample position was ~8 μm at full width at half maximum. The emitted SHG wave was collected by an objective lens with a larger NA (50×, NA = 0.42) and passed through an analyzing WGP. After passing through the WGP, the SHG wave was spectrally filtered by short- and long-pass filters and imaged with a complementary metal-oxide semiconductor camera. The analyzing WGP was rotated to detect the component of the SHG intensity at an angle of φ relative to the *y* axis. The SHG spectrum shown in Fig. 1E was detected by a spectrometer equipped with a charge-coupled device camera. The spectrum was measured without an analyzing WGP and using short-pass filters. All the measurements were performed under vacuum conditions at room temperature.

The SHG-CD signal was derived from the relation $\left(I_{\sigma^{+}}^{2\omega }-I_{\sigma^{-}}^{2\omega }\right)/\left(I_{\sigma^{+}}^{2\omega }+I_{\sigma^{-}}^{2\omega }\right)$, where $I_{\sigma^{+}}^{2\omega }$ and $I_{\sigma^{-}}^{2\omega }$ are the total SHG intensities under σ^+^ and σ^−^ circularly polarized FWs, respectively. $I_{\sigma^{+}}^{2\omega }$ ($I_{\sigma^{-}}^{2\omega }$) was obtained by integrating the measured $I_{\sigma^{+}}^{2\omega }\left(\varphi \right)$ [$I_{\sigma^{-}}^{2\omega }\left(\varphi \right)$] over φ = 0 to φ = 2π or by directly measuring the total SHG intensity for σ^+^ (σ^−^) circularly polarized FW with the analyzing WGP removed from the optical path. The SHG-CD signals shown in Figs. 3 (E and F) and 4F were directly measured without the analyzing WGP.

In the electrical switching measurements, the voltage pulses were generated by a function generator (WF 1974, NF Corporation) and amplified by a high-speed bipolar amplifier (HSA 4051, NF Corporation). The output of the bipolar amplifier was connected to the two contact pads of the interdigitated electrodes. The voltage pulses from the function generator were computer controlled. The samples were electrically poled by sequentially changing the amplitude of the voltage pulse *V* as follows: *V* started at −6 V, decreased to −138 V, increased to +138 V, and decreased to +6 V in increments of 12 V. The SHG signals for each *V* were measured after applying five consecutive voltage pulses with the same amplitude of *V*, where the interval between each pulse was 1 s. The laser beam was blocked during the application of the voltage pulses.

### Polarization dependence of the SHG intensity from as-prepared samples

SHG is characterized by the second-order nonlinear optical susceptibility $\chi_{\mathrm{ijk}}^{\left(2\right)}$, where *i*, *j*, and *k* represent the components of the laboratory coordinate system (*x*, *y*, and *z*). The SHG intensity *I*^2ω^ is proportional to the square of the second-order nonlinear polarization **P**^(2)^(2ω) with twice the frequency of the incident FW, where $P_{i}^{\left(2\right)}\left(2\omega \right)=\varepsilon_{0}\sum_{j,k}\chi_{\mathrm{ijk}}^{\left(2\right)}E_{j}\left(\omega \right)E_{k}\left(\omega \right)$ [ε_0_ is the vacuum permittivity, and *E*(ω) is the electric field of the FW]. Because the relation $\chi_{\mathrm{ijk}}^{\left(2\right)}=\chi_{\mathrm{ikj}}^{\left(2\right)}$ always holds for the SHG process, the nonlinear polarization can be simply represented by a 3 × 6 second-rank tensor *d_nm_* (*n* and *m* are integers from 1 to 3 and from 1 to 6, respectively) (*46*, *47*)

$$\left[\begin{array}{c} P_{x}^{\left(2\right)}\left(2\omega \right)\\ P_{y}^{\left(2\right)}\left(2\omega \right)\\ P_{z}^{\left(2\right)}\left(2\omega \right) \end{array}\right]=2\varepsilon_{0}\left(\begin{array}{cccccc} d_{11} & d_{12} & d_{13} & d_{14} & d_{15} & d_{16}\\ d_{21} & d_{22} & d_{23} & d_{24} & d_{25} & d_{26}\\ d_{31} & d_{32} & d_{33} & d_{34} & d_{35} & d_{36} \end{array}\right)\left[\begin{array}{c} E_{x}{\left(\omega \right)}^{2}\\ E_{y}{\left(\omega \right)}^{2}\\ E_{z}{\left(\omega \right)}^{2}\\ 2E_{y}\left(\omega \right)E_{z}\left(\omega \right)\\ 2E_{x}\left(\omega \right)E_{z}\left(\omega \right)\\ 2E_{x}\left(\omega \right)E_{y}\left(\omega \right) \end{array}\right]$$

In our experiments, the FWs propagated along the *x* direction, i.e., *E_x_*(ω) = 0, and impinged on the sample surface at normal incidence. Therefore, we neglected $P_{x}^{\left(2\right)}\left(2\omega \right)$.

For materials with *mm*2 point group symmetry, which have one twofold rotation axis along the *z* direction and two mirror planes parallel to the *yz* and *xz* planes, there are five independent nonvanishing elements of *d_nm_*: *d*_15_, *d*_24_, *d*_31_, *d*_32_, and *d*_33_ (*46*, *47*). When the crystallographic *b* and *c* axes of the (BA)_2_(EA)_2_Pb_3_I_10_ flakes are parallel to the *y* and *z* axes, respectively, the nonlinear polarization emitted from the flakes can be written using three independent nonvanishing *d_nm_*

$$\left[\begin{array}{c} P_{y}^{\left(2\right)}\left(2\omega \right)\\ P_{z}^{\left(2\right)}\left(2\omega \right) \end{array}\right]=2\varepsilon_{0}\left[\begin{array}{c} 2d_{24}E_{y}\left(\omega \right)E_{z}\left(\omega \right)\\ d_{32}E_{y}{\left(\omega \right)}^{2}+d_{33}E_{z}{\left(\omega \right)}^{2} \end{array}\right]$$ (5)

where $d_{24}=\frac{1}{2}\chi_{\mathrm{yyz}}^{\left(2\right)}=\frac{1}{2}\chi_{\mathrm{yzy}}^{\left(2\right)}$, $d_{32}=\frac{1}{2}\chi_{\mathrm{zyy}}^{\left(2\right)}$, and $d_{33}=\frac{1}{2}\chi_{\mathrm{zzz}}^{\left(2\right)}$. The electric field components of the FWs are [*E_y_*(ω), *E_z_*(ω)] = (*E*_0_ cosθ, *E*_0_ sinθ) for the SHG-RA measurements and $\left[E_{y}\left(\omega \right),E_{z}\left(\omega \right)\right]=\frac{E_{0}}{\sqrt{2}}\left(1,\pm i\right)$ for the SHG-CD measurements, where + (−) sign is for σ^+^ (σ^−^) circular polarization.

The emitted SHG wave passes through the analyzing WGP, and the detected SHG intensity is given by

$$\begin{array}{c} I^{2\omega }\propto {\left|\left(\begin{array}{cc} {\text{cos}}^{2}\varphi & \text{sin}\varphi \text{cos}\varphi \\ \text{sin}\varphi \text{cos}\varphi & {\text{sin}}^{2}\varphi \end{array}\right),\left[\begin{array}{c} P_{y}^{\left(2\right)}\left(2\omega \right)\\ P_{z}^{\left(2\right)}\left(2\omega \right) \end{array}\right]\right|}^{2}\\ ={\left|P_{y}^{\left(2\right)}\left(2\omega \right){\text{cos}}^{2}\varphi +P_{z}^{\left(2\right)}\left(2\omega \right)\text{sin}\varphi \text{cos}\varphi \right|}^{2}\\ +{\left|P_{y}^{\left(2\right)}\left(2\omega \right)\text{sin}\varphi \text{cos}\varphi +P_{z}^{\left(2\right)}\left(2\omega \right){\text{sin}}^{2}\varphi \right|}^{2} \end{array}$$ (6)

Using Eqs. 5 and 6, we can derive expressions for $I_{\text{para}}^{2\omega }\left(\theta \right)$, $I_{\text{perp}}^{2\omega }\left(\theta \right)$, $I_{\sigma^{+}}^{2\omega }\left(\varphi \right)$, and $I_{\sigma^{-}}^{2\omega }\left(\varphi \right)$ for samples with the *b* (*c*) axis rotated from the *y* (*z*) axis by θ*_b_*. $I_{\text{para}}^{2\omega }\left(\theta \right)$ and $I_{\text{perp}}^{2\omega }\left(\theta \right)$ are obtained by setting φ to θ and θ + 90°, respectively, and are found to be proportional to

$$\left({\varepsilon_{0}}^{2}{|E_{0}|}^{4}/2\right)\times \left\{A+B\text{cos}\left[2\left(\theta -\theta_{b}\right)\right]+C\text{cos}\left[4\left(\theta -\theta_{b}\right)\right]+D\text{cos}\left[6\left(\theta -\theta_{b}\right)\right]\right\}$$ (7)

with $A={|\alpha |}^{2}+\frac{{|\beta |}^{2}}{2}-\text{Re}\left\{{\alpha \beta }^{*}\right\}$, $B=2\text{Re}\left\{{\alpha \beta }^{*}\right\}-{|\alpha |}^{2}-\frac{3{|\beta |}^{2}}{4}$, $C=\frac{{|\beta |}^{2}}{4}-\text{Re}\left\{{\alpha \beta }^{*}\right\}$, and $D=-\frac{{|\beta |}^{2}}{4}$ (α = 2*d*_24_ + *d*_32_ + *d*_33_ and β = 2*d*_24_ + *d*_32_ − *d*_33_) for $I_{\text{para}}^{2\omega }\left(\theta \right)$ and $A={|\gamma |}^{2}+\frac{{|\beta |}^{2}}{2}-\text{Re}\left\{{\gamma \beta }^{*}\right\}$, $B={|\gamma |}^{2}-2\text{Re}\left\{{\gamma \beta }^{*}\right\}+\frac{3{|\beta |}^{2}}{4}$, $C=\frac{{|\beta |}^{2}}{2}-\text{Re}\left\{{\gamma \beta }^{*}\right\}$, and $D=\frac{{|\beta |}^{2}}{4}$ (β = 2*d*_24_ + *d*_32_ − *d*_33_ and γ = 2*d*_24_ − *d*_32_ − *d*_33_) for $I_{\text{perp}}^{2\omega }\left(\theta \right)$. In addition, we find that $I_{\sigma^{+}}^{2\omega }\left(\varphi \right)$ and $I_{\sigma^{-}}^{2\omega }\left(\varphi \right)$ are proportional to

$$\left({\varepsilon_{0}}^{2}{|E_{0}|}^{4}\right)\times \left\{E+F\text{cos}\left[2\left(\varphi -\theta_{b}\right)\right]\pm G\text{sin}\left[2\left(\varphi -\theta_{b}\right)\right]\right\}$$ (8)

where + (−) sign is for $I_{\sigma^{+}}^{2\omega }\left(\varphi \right)$ [$I_{\sigma^{-}}^{2\omega }\left(\varphi \right)$] and $E=2{|d_{24}|}^{2}+\frac{{|d_{32}-d_{33}|}^{2}}{2}$, $F=2{|d_{24}|}^{2}-\frac{{|d_{32}-d_{33}|}^{2}}{2}$, and *G* = Re {2*id*_24_(*d*_32_ − *d*_33_)*}. Note that Eq. 8 shows the absence of SHG-CD because the angle-integrated SHG intensities under σ^+^ and σ^−^ circularly polarized FWs are equal.

From Eqs. 7 and 8, we obtain the following relations

$$\begin{array}{c} I_{\text{para}}^{2\omega }\left(\theta_{b}\right)=0\\ I_{\text{perp}}^{2\omega }\left(\theta_{b}\right)\propto 4{\varepsilon_{0}}^{2}{|d_{32}|}^{2}{\left(I^{\omega }\right)}^{2}\\ I_{\text{para}}^{2\omega }\left(\theta_{c}\right)\propto 4{\varepsilon_{0}}^{2}{|d_{33}|}^{2}{\left(I^{\omega }\right)}^{2}\\ I_{\text{perp}}^{2\omega }\left(\theta_{c}\right)=0\\ I_{\sigma^{+}}^{2\omega }\left(\theta_{b}\right)=I_{\sigma^{-}}^{2\omega }\left(\theta_{b}\right)\propto 4{\varepsilon_{0}}^{2}{|d_{24}|}^{2}{\left(I^{\omega }\right)}^{2} \end{array}$$ (9)

where *I*^ω^ is the FW intensity. The spatial maps of ∣*d*_24_∣/∣*d*_33_∣ and ∣*d*_32_∣/∣*d*_33_∣ can be obtained by fitting $I_{\text{para}}^{2\omega }\left(\theta \right)$ and $I_{\text{perp}}^{2\omega }\left(\theta \right)$ with Eq. 1 and $I_{\sigma^{+}}^{2\omega }\left(\varphi \right)$ and $I_{\sigma^{-}}^{2\omega }\left(\varphi \right)$ with Eq. 2 and applying Eq. 9 to the fitting results at each spatial position (Fig. 2, F and G).

### Polarization dependence of the SHG intensity from electrically poled samples

Among the lower symmetry subgroups of point group *mm*2, i.e., point groups 1, 2, and *m*, point group 2 and point group *m* with one mirror plane perpendicular to the *yz* plane give the same polarization dependence as expected for point group *mm*2 under our experimental conditions. In contrast, we find that the other subgroups, i.e., point group 1 and point group *m* with one mirror plane parallel to the *yz* plane, give different polarization dependence from that expected for point group *mm*2 and can explain the observed polarization dependence of the SHG intensity induced by electrical poling. The nonlinear polarization in such lower symmetry point groups with no in-plane mirror symmetry (no mirror planes perpendicular to the *yz* plane) is given by

$$\left[\begin{array}{c} P_{y}^{\left(2\right)}\left(2\omega \right)\\ P_{z}^{\left(2\right)}\left(2\omega \right) \end{array}\right]=2\varepsilon_{0}\left[\begin{array}{c} d_{22}E_{y}{\left(\omega \right)}^{2}+d_{23}E_{z}{\left(\omega \right)}^{2}+2d_{24}E_{y}\left(\omega \right)E_{z}\left(\omega \right)\\ d_{32}E_{y}{\left(\omega \right)}^{2}+d_{33}E_{z}{\left(\omega \right)}^{2}+2d_{34}E_{y}\left(\omega \right)E_{z}\left(\omega \right) \end{array}\right]$$ (10)

where $d_{22}=\frac{1}{2}\chi_{\mathrm{yyy}}^{\left(2\right)}$, $d_{23}=\frac{1}{2}\chi_{\mathrm{yzz}}^{\left(2\right)}$, $d_{24}=\frac{1}{2}\chi_{\mathrm{yyz}}^{\left(2\right)}=\frac{1}{2}\chi_{\mathrm{yzy}}^{\left(2\right)}$, $d_{32}=\frac{1}{2}\chi_{\mathrm{zyy}}^{\left(2\right)}$, $d_{33}=\frac{1}{2}\chi_{\mathrm{zzz}}^{\left(2\right)}$, and $d_{34}=\frac{1}{2}\chi_{\mathrm{zyz}}^{\left(2\right)}=\frac{1}{2}\chi_{\mathrm{zzy}}^{\left(2\right)}$. Using Eq. 10, we find that $I_{\text{para}}^{2\omega }\left(\theta \right)$ and $I_{\text{perp}}^{2\omega }\left(\theta \right)$ are proportional to

$$\begin{array}{c} \left({\varepsilon_{0}}^{2}{|E_{0}|}^{4}\right)\times \left\{A\prime +B\prime \text{cos}\left[2\left(\theta -\theta_{b}^{\prime }\right)\right]+C\prime \text{cos}\left[4\left(\theta -\theta_{b}^{\prime }\right)\right]+D\prime \text{cos}\left[6\left(\theta -\theta_{b}^{\prime }\right)\right]+\right.\\ \left.E\prime \text{sin}\left[2\left(\theta -\theta_{b}^{\prime }\right)\right]+F\prime \text{sin}\left[4\left(\theta -\theta_{b}^{\prime }\right)\right]+G\prime \text{sin}\left[6\left(\theta -\theta_{b}^{\prime }\right)\right]\right\} \end{array}$$ (11)

where the coefficients of *A*′ to *G*′ are lengthy functions of *d*_22_, *d*_23_, *d*_24_, *d*_32_, *d*_33_, and *d*_34_ and take different values for $I_{\text{para}}^{2\omega }\left(\theta \right)$ and $I_{\text{perp}}^{2\omega }\left(\theta \right)$. We also find that $I_{\sigma^{+}}^{2\omega }\left(\varphi \right)$ and $I_{\sigma^{-}}^{2\omega }\left(\varphi \right)$ are proportional to

$$\left({\varepsilon_{0}}^{2}{|E_{0}|}^{4}\right)\times \left\{H\prime +I\prime \text{cos}\left[2\left(\varphi -\theta_{b}^{\prime }\right)\right]+J\prime \text{sin}\left[2\left(\varphi -\theta_{b}^{\prime }\right)\right]\right\}$$ (12)

with $H\prime =\frac{{|\alpha^{\prime }|}^{2}+{|\beta^{\prime }|}^{2}}{2}$, $I\prime =\frac{{|\alpha^{\prime }|}^{2}-{|\beta^{\prime }|}^{2}}{2}$, and *J*′ = Re {α′β′*}, where α′ = *d*_22_ − *d*_23_ + 2*id*_24_ and β′ = *d*_32_ − *d*_33_ + 2*id*_34_ for $I_{\sigma^{+}}^{2\omega }\left(\varphi \right)$ and α′ = *d*_22_ − *d*_23_ − 2*id*_24_ and β′ = *d*_32_ − *d*_33_ − 2*id*_34_ for $I_{\sigma^{-}}^{2\omega }\left(\varphi \right)$.

From Eq. 11, we obtain the following relations

$$\begin{array}{c} I_{\text{para}}^{2\omega }\left(\theta_{b}^{\prime }\right)\propto 4{\varepsilon_{0}}^{2}{|d_{22}|}^{2}{\left(I^{\omega }\right)}^{2}\\ I_{\text{perp}}^{2\omega }\left(\theta_{b}^{\prime }\right)\propto 4{\varepsilon_{0}}^{2}{|d_{32}|}^{2}{\left(I^{\omega }\right)}^{2}\\ I_{\text{para}}^{2\omega }\left(\theta_{c}^{\prime }\right)\propto 4{\varepsilon_{0}}^{2}{|d_{33}|}^{2}{\left(I^{\omega }\right)}^{2}\\ I_{\text{perp}}^{2\omega }\left(\theta_{c}^{\prime }\right)\propto 4{\varepsilon_{0}}^{2}{|d_{23}|}^{2}{\left(I^{\omega }\right)}^{2} \end{array}$$

with $\theta_{c}^{\prime }=\theta_{b}^{\prime }+90{}^{\circ}$. Furthermore, Eq. 12 gives

$$\begin{array}{c} I_{\sigma^{+}}^{2\omega }\left(\theta_{b}^{\prime }\right)\propto {\varepsilon_{0}}^{2}{|d_{22}-d_{23}+2id_{24}|}^{2}{\left(I^{\omega }\right)}^{2}\\ I_{\sigma^{-}}^{2\omega }\left(\theta_{b}^{\prime }\right)\propto {\varepsilon_{0}}^{2}{|d_{22}-d_{23}-2id_{24}|}^{2}{\left(I^{\omega }\right)}^{2}\\ I_{\sigma^{+}}^{2\omega }\left(\theta_{c}^{\prime }\right)\propto {\varepsilon_{0}}^{2}{|d_{32}-d_{33}+2id_{34}|}^{2}{\left(I^{\omega }\right)}^{2}\\ I_{\sigma^{-}}^{2\omega }\left(\theta_{c}^{\prime }\right)\propto {\varepsilon_{0}}^{2}{|d_{32}-d_{33}-2id_{34}|}^{2}{\left(I^{\omega }\right)}^{2} \end{array}$$ (13)

When ∣*d*_23_/*d*_22_∣ ≪ 1 and ∣*d*_32_/*d*_33_∣ ≪ 1 hold, Eq. 13 gives

$$\begin{array}{c} I_{\sigma^{+}}^{2\omega }\left(\theta_{b}^{\prime }\right)+I_{\sigma^{-}}^{2\omega }\left(\theta_{b}^{\prime }\right)\propto 2{\varepsilon_{0}}^{2}{|d_{22}|}^{2}\left\{1+4{|d_{24}/d_{22}|}^{2}\right\}{\left(I^{\omega }\right)}^{2}\\ I_{\sigma^{+}}^{2\omega }\left(\theta_{c}^{\prime }\right)+I_{\sigma^{-}}^{2\omega }\left(\theta_{c}^{\prime }\right)\propto 2{\varepsilon_{0}}^{2}{|d_{33}|}^{2}\left\{1+4{|d_{34}/d_{33}|}^{2}\right\}{\left(I^{\omega }\right)}^{2} \end{array}$$

and these relations lead to

$$\begin{array}{c} I_{\sigma^{+}}^{2\omega }\left(\theta_{b}^{\prime }\right)+I_{\sigma^{-}}^{2\omega }\left(\theta_{b}^{\prime }\right)-\frac{I_{\text{para}}^{2\omega }\left(\theta_{b}^{\prime }\right)}{2}\propto 8{\varepsilon_{0}}^{2}{|d_{24}|}^{2}{\left(I^{\omega }\right)}^{2}\\ I_{\sigma^{+}}^{2\omega }\left(\theta_{c}^{\prime }\right)+I_{\sigma^{-}}^{2\omega }\left(\theta_{c}^{\prime }\right)-\frac{I_{\text{para}}^{2\omega }\left(\theta_{c}^{\prime }\right)}{2}\propto 8{\varepsilon_{0}}^{2}{|d_{34}|}^{2}{\left(I^{\omega }\right)}^{2} \end{array}$$

### SHG from the ferroelectric multidomain structure

As shown in Fig. 1B, the spontaneous polarization of ferroelectric (BA)_2_(EA)_2_Pb_3_I_10_ can be in four equivalent directions. Here, we calculate the second-order nonlinear polarization generated from a multidomain structure consisting of four domain variants with each spontaneous polarization directed in one of the four equivalent directions, where each domain variant has *mm*2 point group symmetry. In the domain variant whose *b* (*c*) axis is parallel to the *y* (*z*) axis (domain 1), the second-order nonlinear polarization, $\left[P_{d1,y}^{\left(2\right)}\left(2\omega \right),P_{d1,z}^{\left(2\right)}\left(2\omega \right)\right]$, is given by Eq. 5. When the spontaneous polarization is antiparallel to that in domain 1, i.e., the *b* (*c*) axis of the domain is antiparallel to the *y* (*z*) axis (domain 2), the nonlinear polarization generated from domain 2, $\left[P_{d2,y}^{\left(2\right)}\left(2\omega \right),P_{d2,z}^{\left(2\right)}\left(2\omega \right)\right]$, is out of phase with that from domain 1, i.e., $\left[P_{d2,y}^{\left(2\right)}\left(2\omega \right),P_{d2,z}^{\left(2\right)}\left(2\omega \right)\right]=-\left[P_{d1,y}^{\left(2\right)}\left(2\omega \right),P_{d1,z}^{\left(2\right)}\left(2\omega \right)\right].$ When the spontaneous polarization is perpendicular to that in domain 1, there are two different directions of the crystallographic axes: The *b* (*c*) axis is parallel (antiparallel) to the *z* (*y*) axis (domain 3), and the *b* (*c*) axis is antiparallel (parallel) to the *z* (*y*) axis (domain 4). The nonlinear polarizations from domains 3 and 4 can be written as

$$\left[\begin{array}{c} P_{d3,y}^{\left(2\right)}\left(2\omega \right)\\ P_{d3,z}^{\left(2\right)}\left(2\omega \right) \end{array}\right]=-\left[\begin{array}{c} P_{d4,y}^{\left(2\right)}\left(2\omega \right)\\ P_{d4,z}^{\left(2\right)}\left(2\omega \right) \end{array}\right]=-2\varepsilon_{0}\left[\begin{array}{c} d_{33}E_{y}{\left(\omega \right)}^{2}+d_{32}E_{z}{\left(\omega \right)}^{2}\\ 2d_{24}E_{y}\left(\omega \right)E_{z}\left(\omega \right) \end{array}\right]$$

Therefore, denoting the area fraction of domain *i* (*i* = 1 to 4) as *A_i_*, where *A*_1_ + *A*_2_ + *A*_3_ + *A*_4_ = 1, we obtain the nonlinear polarization from the four domain variants as follows (*46*, *52*)

$$\begin{array}{c} \left[\begin{array}{c} P_{y}^{\left(2\right)}\left(2\omega \right)\\ P_{z}^{\left(2\right)}\left(2\omega \right) \end{array}\right]=A_{1}\left[\begin{array}{c} P_{d1,y}^{\left(2\right)}\left(2\omega \right)\\ P_{d1,z}^{\left(2\right)}\left(2\omega \right) \end{array}\right]+A_{2}\left[\begin{array}{c} P_{d2,y}^{\left(2\right)}\left(2\omega \right)\\ P_{d2,z}^{\left(2\right)}\left(2\omega \right) \end{array}\right]\\ +\left(\begin{array}{cc} e^{i\Gamma_{y}} & 0\\ 0 & e^{i\Gamma_{z}} \end{array}\right)\left\{A_{3}\left[\begin{array}{c} P_{d3,y}^{\left(2\right)}\left(2\omega \right)\\ P_{d3,z}^{\left(2\right)}\left(2\omega \right) \end{array}\right]+A_{4}\left[\begin{array}{c} P_{d4,y}^{\left(2\right)}\left(2\omega \right)\\ P_{d4,z}^{\left(2\right)}\left(2\omega \right) \end{array}\right]\right\}\\ =2\varepsilon_{0}\left[\begin{array}{c} -e^{i\Gamma_{y}}\delta A_{90{}^{\circ}}d_{33}E_{y}{\left(\omega \right)}^{2}-e^{i\Gamma_{y}}\delta A_{90{}^{\circ}}d_{32}E_{z}{\left(\omega \right)}^{2}+2\delta A_{0{}^{\circ}}d_{24}E_{y}\left(\omega \right)E_{z}\left(\omega \right)\\ {\delta A}_{0{}^{\circ}}d_{32}E_{y}{\left(\omega \right)}^{2}+{\delta A}_{0{}^{\circ}}d_{33}E_{z}{\left(\omega \right)}^{2}-2e^{i\Gamma_{z}}\delta A_{90{}^{\circ}}d_{24}E_{y}\left(\omega \right)E_{z}\left(\omega \right) \end{array}\right] \end{array}$$ (14)

where δ*A*_0°_ = *A*_1_ − *A*_2_ and δ*A*_90°_ = *A*_3_ − *A*_4_, which reflect the magnitude of the net spontaneous polarization along the *c* axis of domains 1 and 2 and domains 3 and 4, respectively. Γ*_y_* and Γ*_z_* are the *y* and *z* components, respectively, of the relative phase between the second-order nonlinear polarizations from the domains with perpendicular spontaneous polarization directions. We find that Eq. 14 shows the same dependence on the polarization of the incident FW as that of Eq. 10; this leads to the following relations: ∣*d*_22_∣^2^, ∣*d*_23_∣^2^, ∣*d*_24_∣^2^, ∣*d*_32_∣^2^, ∣*d*_33_∣^2^, and ∣*d*_34_∣^2^ in Eq. 10 correspond to ∣δ*A*_90°_∣^2^∣*d*_33_∣^2^, ∣δ*A*_90°_∣^2^∣*d*_32_∣^2^, ∣δ*A*_0°_∣^2^∣*d*_24_∣^2^, ∣δ*A*_0°_∣^2^∣*d*_32_∣^2^, ∣δ*A*_0°_∣^2^∣*d*_33_∣^2^, and ∣δ*A*_90°_∣^2^∣*d*_24_∣^2^ in Eq. 14.

By using Eq. 14, SHG-CD can be written as

$$\frac{I_{\sigma^{+}}^{2\omega }-I_{\sigma^{-}}^{2\omega }}{I_{\sigma^{+}}^{2\omega }+I_{\sigma^{-}}^{2\omega }}=\frac{4{\delta A}_{0{}^{\circ}}{\delta A}_{90{}^{\circ}}\text{Im}\left\{\left(d_{32}-d_{33}){d_{24}}^{*}(e^{i\Gamma_{y}}-e^{-i\Gamma_{z}}\right)\right\}}{\left(|{\delta A}_{0{}^{\circ}}{|}^{2}+|{\delta A}_{90{}^{\circ}}{|}^{2}\right)\left(4{|d_{24}|}^{2}+{|d_{32}-d_{33}|}^{2}\right)}$$

This shows that the change of the sign of SHG-CD is related to the modulation of δ*A*_0°_, δ*A*_90°_, Γ*_y_*, and Γ*_z_* by assuming that the nonlinear optical tensor elements do not change by applying voltage pulses. In our experiments, the sign of δ*A*_0°_ (δ*A*_90°_), i.e., the direction of the net spontaneous polarization related to δ*A*_0°_ (δ*A*_90°_), does not change during the electrical poling (Supplementary Text S3). Therefore, the electrically induced changes of Γ*_y_* and Γ*_z_* are the origin of the observed sign change of SHG-CD. Using the refractive indices at 2ω along the *y* (*z*) axis in the domains related to δ*A*_0°_ and δ*A*_90°_, $n_{y\left(z\right),0{}^{\circ}}^{2\omega }$ and $n_{y\left(z\right),90{}^{\circ}}^{2\omega }$, respectively, we can write Γ*_y_* and Γ*_z_* as $\left(2\omega l/c\right)\left(n_{y,0{}^{\circ}}^{2\omega }-n_{y,90{}^{\circ}}^{2\omega }\right)$ and $\left(2\omega l/c\right)\left(n_{z,0{}^{\circ}}^{2\omega }-n_{z,90{}^{\circ}}^{2\omega }\right)$, respectively (*46*). Here, *l* is the thickness of the sample and *c* is the speed of light. Therefore, the electrical modulation of Γ*_y_* and Γ*_z_* would be attributed to the electrically induced change of the effective Pockels coefficient depending on the ferroelectric domain configuration (*53*).

## Supplementary Materials

### This PDF file includes:

Supplementary Texts S1 to S5 Figs. S1 to S11 References
