# Supplementary material for: Electrically switchable chiral nonlinear optics in an achiral ferroelectric 2D van der Waals halide perovskite
Source: Sci Adv. 2024 Nov 13;10(46):eadq5521. doi: 10.1126/sciadv.adq5521 (PMC12697557; doi:10.1126/sciadv.adq5521)
Supplement: Supplementary file 1 — Supplementary Texts S1 to S5 Figs. S1 to S11 References [file sciadv.adq5521_sm.pdf]

Science Advances
AAAS

Supplementary Materials for

Electrically switchable chiral nonlinear optics in an achiral ferroelectric 2D van der Waals halide perovskite

Go Yumoto *et al.*

Corresponding author: Go Yumoto, yumoto@issp.u-tokyo.ac.jp; Yoshihiko Kanemitsu, kanemitu@scl.kyoto-u.ac.jp

Sci. Adv. **10**, eadq5521 (2024)
DOI: 10.1126/sciadv.adq5521

This PDF file includes:

Supplementary Text S1 to S5
Figs. S1 to S11
References

Supplementary Text

S1: Estimation of sample thickness

The thickness of the exfoliated flakes was estimated by using absorption spectra and atomic force microscopy. Figure S2A shows the absorbance spectrum of an exfoliated $(BA)_2(EA)_2Pb_3I_{10}$ flake at room temperature, which was obtained by measuring the reflectance and transmittance spectra as reported in previous work (*16*, *43*, *54*). As can be seen from Fig. S2A, the interference pattern appearing in the reflectance (R) and transmittance (T) spectra (*4*, *55*) is not observed in the absorbance spectrum because the absorbance is given by $-\log(T/(1-R))$ and the interference is compensated. The thickness of this flake, as measured by atomic force microscopy, was 470 nm (Fig. S2B). Having determined the relation between the absorbance and the crystal thickness, we can estimate the thicknesses of different flakes simply by measuring their absorbance spectra because the absorbance is proportional to the crystal thickness.

S2: Reproducibility of SHG-CD switching for different samples

To ensure the reproducibility of the electrical switching of SHG-CD demonstrated in the main text, we performed polarization-resolved SHG imaging on different exfoliated flakes on gold interdigitated electrodes. For the crystal flake shown in Fig. S7A, we measured $I^{2\omega}_{\mathrm{para}}(\theta)$ and $I^{2\omega}_{\mathrm{perp}}(\theta)$ (Fig. S7B) and $I^{2\omega}_{\sigma^+}(\varphi)$ and $I^{2\omega}_{\sigma^-}(\varphi)$ (Fig. S7C) before applying the voltage pulses. The polarization dependences of the SHG signals were well reproduced under the assumption of *mm*2 point group symmetry and the orientation angle of the *c*-axis was estimated to be 38 deg relative to the *y*-axis, which corresponds to the angle between the *c*-axis and the direction parallel to the electrodes of 53 deg. Figure S7D illustrates a series of the SHG-CD maps acquired by applying sequential voltage pulses to the sample. As indicated in the main text, the SHG-CD signals were electrically induced and switched in this flake. The hysteretic nature and large maximum of the SHG-CD (0.85) were also observed (Fig. S7E).

S3: Determination of the net-spontaneous-polarization direction

The directions of the net spontaneous polarizations can be determined from the voltage dependences of the SHG intensities shown in Fig. 4C in the main text. The SHG intensities proportional to $|d_{22}|^2$ ($|\delta A_{90\mathrm{deg}}|^2|d_{33}|^2$) and $|d_{34}|^2$ ($|\delta A_{90\mathrm{deg}}|^2|d_{24}|^2$) increase with decreasing voltage in the negative direction. This shows that the net polarization of domains 3 and 4 is directed at an angle of 223 deg (not 43 deg) relative to the *y*-axis for negative voltages. This is because in the experimental configurations shown in Fig. 3A in the main text, the voltage pulses with negative (positive) amplitudes orient the spontaneous polarization in the negative (positive) *y*-direction. When the voltage is increased to +138 V and then decreased back to 0 V, these SHG intensities vanish and do not recover. This suppression of SHG originates from the destructive interference of the SHG signals from domains 3 and 4 with $\delta A_{90\mathrm{deg}} = 0$. Therefore, we found that the direction of the net spontaneous polarization of domains 3 and 4 is not reversed and stays at an angle of 223 deg during electrical poling. In the same way, the direction of the net spontaneous polarization of domains 1 and 2 was determined to be at an angle of 313 deg.

S4: Recovery of *mm*2 point group symmetry

When SHG-CD ~ 0 and $R_{0-90\mathrm{deg}} \sim \pm 1$, only the domain related to $\delta A_{0\mathrm{deg}}$ or $\delta A_{90\mathrm{deg}}$ has a net spontaneous polarization and *mm*2 point group symmetry should be recovered. Figure S9A shows the maps of SHG-CD and $R_{0-90\mathrm{deg}}$ for $V = -138$ and +138 V. In the boxed region in Fig.

S9A, we obtained an average SHG-CD of 0.05 ± 0.03 for $V = -138$ V and −0.01 ± 0.02 for $V =$ +138 V, i.e., negligible SHG-CD for both voltages. On the other hand, the average $R_{0-90\mathrm{deg}}$ was 0.95 ± 0.08 for $V = -138$ V and −0.95 ± 0.08 for $V = +138$ V. Therefore, the boxed region for $V = -138$ V (+138 V) corresponds to the case where SHG-CD ~ 0 and $R_{0-90\mathrm{deg}} \sim +1\ (-1)$; i.e., only the domain related to $\delta A_{90\mathrm{deg}}$ ($\delta A_{0\mathrm{deg}}$) has a net spontaneous polarization.

Figure S9B shows $I^{2\omega}_{\mathrm{para}}(\theta)$ and $I^{2\omega}_{\mathrm{perp}}(\theta)$ in the boxed region for $V = -138$ and +138 V. We found that all the SHG-RA patterns are well reproduced under the assumption of *mm*2 point group symmetry and that the orientation of the symmetric axis of the SHG-RA patterns, i.e., the *c*-axis, for $V = -138$ V was different from that for $V = +138$ V. By fitting to the data using Eq. 1 in the main text, we estimated the orientation angle of the *c*-axis relative to the *y*-axis to be 44 deg for $V = -138$ V and 132 deg for $V = +138$ V, which are in good agreement with the respective orientation angles of the *c*-axis of the domains related to $\delta A_{90\mathrm{deg}}$ (43 deg) and $\delta A_{0\mathrm{deg}}$ (133 deg). These results further support our interpretation of the origin of the electrically switchable SHG-CD.

S5: Electrical poling of sample with spontaneous polarization oriented perpendicular to the electrodes

The importance of coexisting domains to the emergence of the nonlinear chiroptical responses was further clarified by performing polarization-resolved SHG imaging on a flake with the spontaneous polarization aligned perpendicular to the electrodes. In such an experimental configuration, the spontaneous polarization parallel to the electrodes cannot be modulated by electrical poling. To perform measurements under these conditions, we used the crystal flake shown in Fig. S10A. Before applying the voltage pulses, we found that the SHG signals from the flake could be well explained by the *mm*2 point group symmetry, and we observed a homogeneous spatial distribution of zero SHG-CD values (Fig. S10B), $|d_{24}|/|d_{33}|$, $|d_{32}|/|d_{33}|$, and θ_c (Fig. S10C). The spatially averaged values were estimated to be $|d_{24}|/|d_{33}|$ = 0.73 ± 0.09, $|d_{32}|/|d_{33}|$ = 0.1 ± 0.2, and θ_c = 7 ± 2 deg. The value of θ_c corresponds to the angle between the *c*-axis and the direction parallel to the electrodes of 84 deg, which shows that the spontaneous polarization before poling is aligned almost perpendicular to the electrodes.

Figure S10D illustrates a series of the SHG-CD maps acquired by applying sequential voltage pulses to the sample. In stark contrast to the results for the crystal flakes whose spontaneous polarizations before poling were neither parallel nor perpendicular to the electrodes (see Fig. 3E in the main text and Fig. S7D), the electrical poling did not induce finite SHG-CD signals and the SHG-CD remained zero at every applied voltage. To further investigate the SHG responses acquired from this sample under sequential voltage pulses, we plotted a series of maps of SHG intensities proportional to $|d_{22}|^2$ (Fig. S10E) and $|d_{33}|^2$ (Fig. S10F) for the 1 or *m* point group symmetries. These SHG intensities were obtained from $I^{2\omega}_{\mathrm{para}}(\theta'_b = \theta'_c - 90\ \mathrm{deg})$ and $I^{2\omega}_{\mathrm{para}}(\theta'_c)$, respectively, with $\theta'_c = 7$ deg which corresponds to θ_c before poling. The figures show no significant SHG signals proportional to $|d_{22}|^2$ for all *V*, while the SHG intensity proportional to $|d_{33}|^2$ is modulated depending on *V*.

These behaviors are clearly seen in the voltage dependences of the SHG signals averaged within the black-boxed region in Fig. S10D (Figs. S10G and H). Figure S10G shows the voltage dependences of $I^{2\omega}_{\sigma^+}$, $I^{2\omega}_{\sigma^-}$, and SHG-CD signals. We found that although the electrical poling modulated $I^{2\omega}_{\sigma^+}$ and $I^{2\omega}_{\sigma^-}$ with hysteretic behavior, the SHG-CD did not change with voltage and remained zero. That the SHG-CD remains zero regardless of the applied voltage indicates that

the electrical poling does not induce the multidomain structure with perpendicular spontaneous polarizations and breaking of the in-plane glide mirror symmetry. Figure S10H, showing the voltage dependence of the SHG intensities proportional to $|d_{22}|^2$ and $|d_{33}|^2$, confirms this conclusion. The electrical poling modulates only the SHG intensity proportional to $|d_{33}|^2$ with hysteretic behavior, while the SHG intensity proportional to $|d_{22}|^2$ does not change with the applied voltage and shows no significant signals. Because the spontaneous polarizations related to $|d_{33}|^2$ and $|d_{22}|^2$ are perpendicular to each other, the voltage dependence of the SHG intensities shows that the multidomain structure with perpendicular spontaneous polarizations does not appear by poling. This can be understood by considering that the spontaneous polarizations related to $|d_{33}|^2$ and $|d_{22}|^2$ are tilted from the direction parallel to the electrodes by 84 and 6 deg, i.e., oriented almost perpendicular and parallel to the electrodes, respectively. Because the spontaneous polarization parallel to the electrodes cannot be modulated by the electrical poling, the SHG intensity proportional to $|d_{22}|^2$ shows no voltage dependence. The simultaneous absences of a finite SHG-CD signal and a multidomain structure in these results further verifies our interpretation of the origin of the electrically switchable nonlinear chiroptical responses.

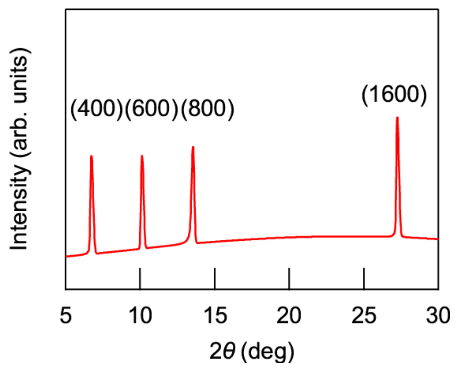

Fig. S1. n = 3 phase purity of $(BA)_2(EA)_2Pb_3I_{10}$ samples. X-ray diffraction spectrum of $(BA)_2(EA)_2Pb_3I_{10}$ crystals, which confirms a phase purity of $n = 3$. The vertical axis is on a log scale.

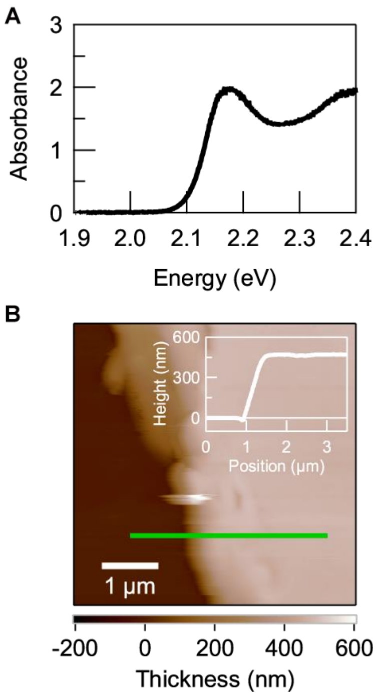

Fig. S2. Absorption spectrum and atomic force microscopy image of an exfoliated $(BA)_2(EA)_2Pb_3I_{10}$ flake. (**A**) Absorbance spectrum of an exfoliated sample. (**B**) Atomic force microscopy image of the same exfoliated sample. The inset is a height profile along the green line in the image. Scale bar, 1 µm.

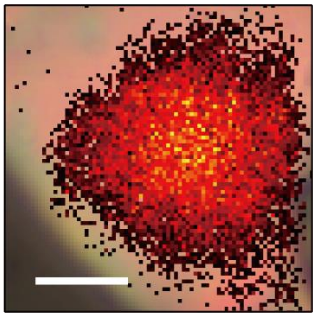

Fig. S3. Optical microscope image overlaid with corresponding SHG intensity map. Optical microscope image of the area marked by the green square in Fig. 2A in the main text, which is overlaid with the SHG intensity map with $\theta = \varphi = 31$ deg corresponding to that shown in Fig. 2B in the main text. Scale bar, 3 μm.

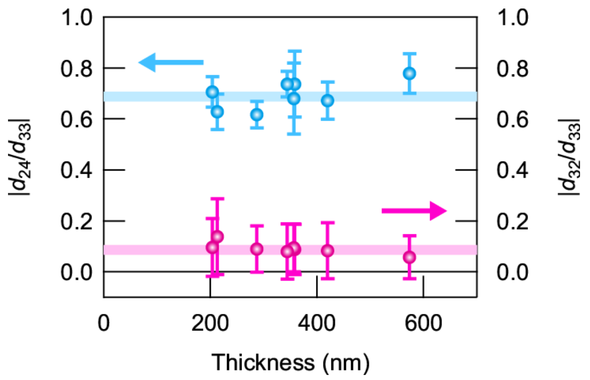

Fig. S4. $|d_{24}|/|d_{33}|$ **and** $|d_{32}|/|d_{33}|$ **estimated in as-prepared (BA)$_2$(EA)$_2$Pb$_3$I$_{10}$ flakes with different thicknesses.** $|d_{24}|/|d_{33}|$ (blue circles; left axis) and $|d_{32}|/|d_{33}|$ (red circles; right axis) as a function of sample thickness. The error bars show the standard deviation of spatially averaged values. The lines are linear least-square fits with a slope fixed to zero.

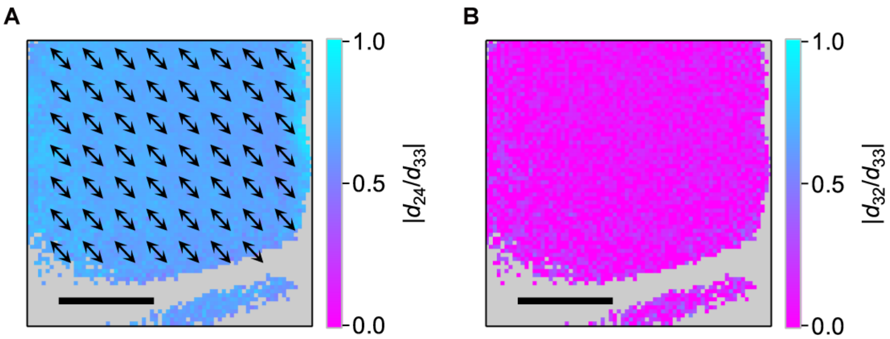

Fig. S5. Spatial distributions of $|d_{24}|/|d_{33}|$, $|d_{32}|/|d_{33}|$, and θ_c in the crystal flake before electrical poling. (**A** and **B**) Maps of $|d_{24}|/|d_{33}|$ (A) and $|d_{32}|/|d_{33}|$ (B) before electrical poling, estimated in the same spatial region shown in Fig. 3B in the main text. The black double-sided arrows in (A) indicate the two possible directions of the *c*-axis at each spatial position. The grey-shaded areas correspond to the regions where the related SHG intensities are weak or not observable. Scale bars, 3 μm.

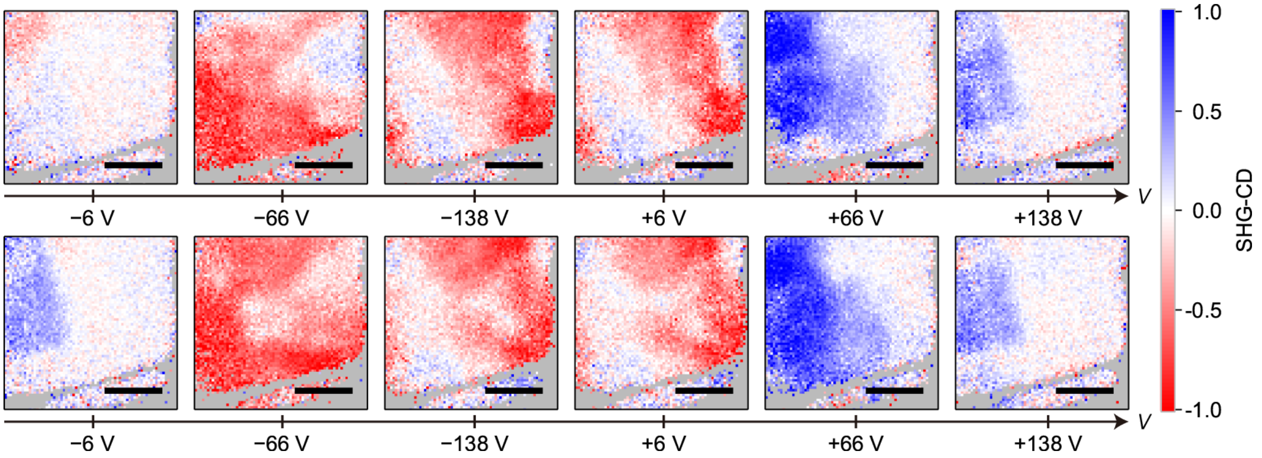

Fig. S6. Electrical switching of the SHG-CD signals for different voltage cycles. A series of maps of SHG-CD after applying voltage pulses with different amplitudes during the poling process. The top panels show SHG-CD maps measured during a single voltage cycle, while the bottom panels show those obtained during the subsequent cycle. The bottom panels correspond to those shown in Fig. 3E in the main text. The grey-shaded areas correspond to the regions where the related SHG intensities are weak or not observable. Scale bars, 3 μm.

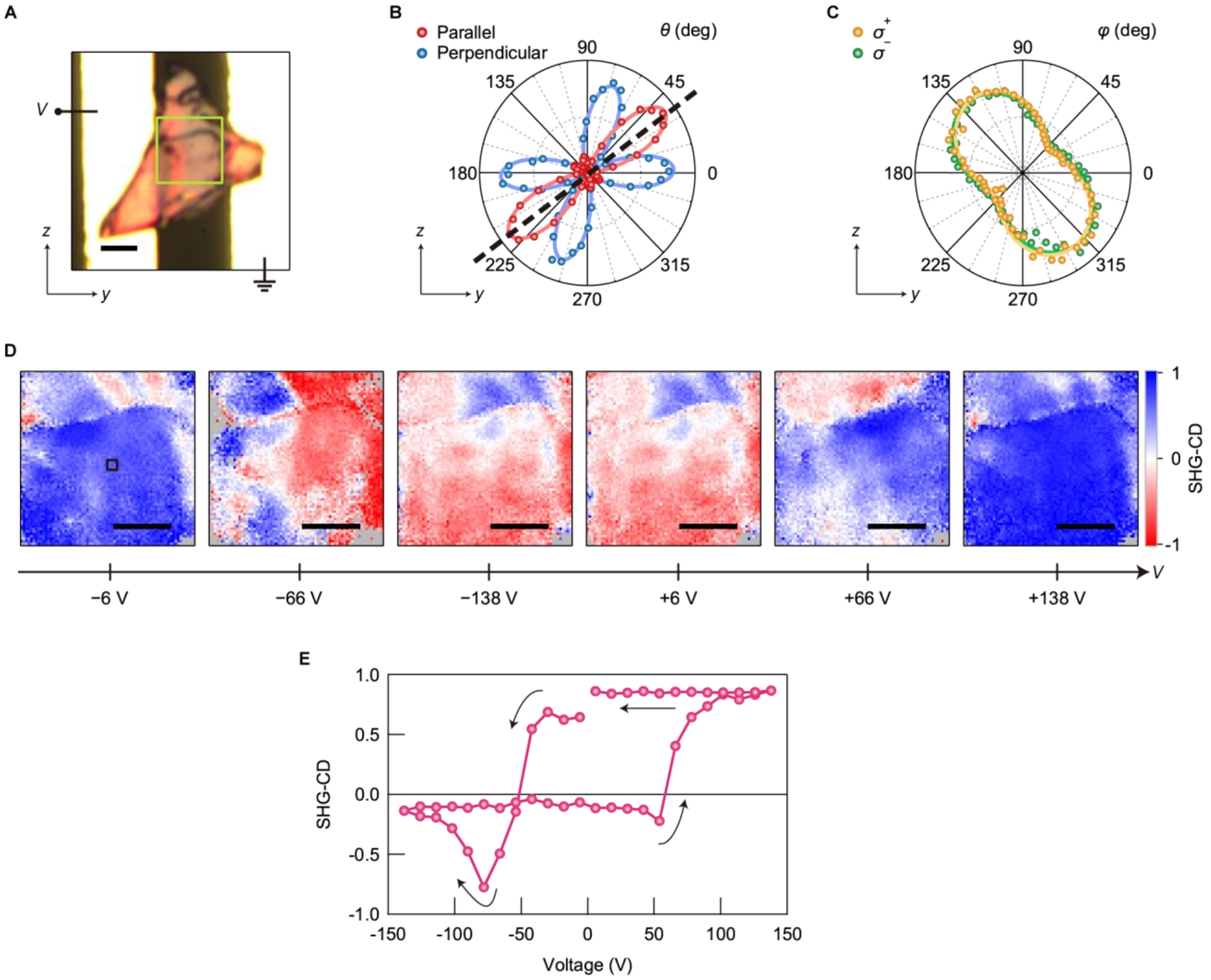

Fig. S7. Electrically switchable chiral SHG in another $(BA)_2(EA)_2Pb_3I_{10}$ flake. (**A**) Optical microscope image of an exfoliated sample on interdigitated electrodes with the *y*- and *z*-axes of the laboratory coordinate system. The right electrode was connected to ground. Scale bar, 5 μm. (**B**) Polar plots of the SHG intensities before poling obtained in the parallel (red circles) and perpendicular (blue circles) configurations of the SHG-RA measurements as a function of θ. The red and blue curves are fits using Eq. 1 in the main text. The black dashed line is along the crystallographic *c*-axis and shows the orientation of the glide mirror plane. (**C**) Polar plots of the SHG intensities before poling with σ^+ (orange circles) and σ^- (green circles) circularly polarized FWs as a function of φ. The orange and green curves are fits using Eq. 2 in the main text. The SHG intensities in (B and C) were obtained by averaging the signals in the area marked by the green square in (A). (**D**) A series of maps of the SHG-CD after applying voltage pulses with different amplitudes during the poling process. The displayed region is the area marked by the green square in (A). The grey-shaded areas correspond to the regions where the related SHG intensities are weak or not observable. Scale bars, 3 μm. (**E**) Hysteresis of the SHG-CD obtained by averaging the signals in the boxed area in the leftmost panel of (D). The arrows indicate the switching sequence.

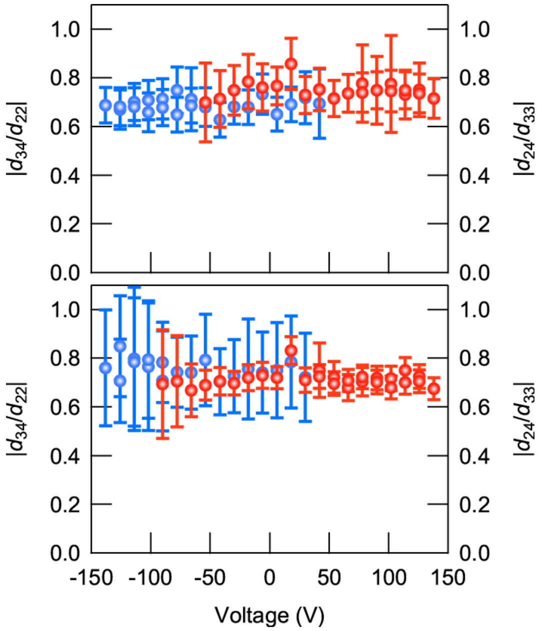

Fig. S8. $|d_{34}|/|d_{22}|$ and $|d_{24}|/|d_{33}|$ during electrical poling. Voltage dependences of $|d_{34}|/|d_{22}|$ (blue circles; left axis) and $|d_{24}|/|d_{33}|$ (red circles; right axis). The top and bottom panels show data obtained by averaging in different areas and the averaging areas correspond to those in Fig. 4C in the main text. $|d_{34}|/|d_{22}|$ ($|d_{24}|/|d_{33}|$) for the voltages where $|d_{22}|$ ($|d_{33}|$) takes small values is not plotted. The error bars show the standard deviation of the spatially averaged values.

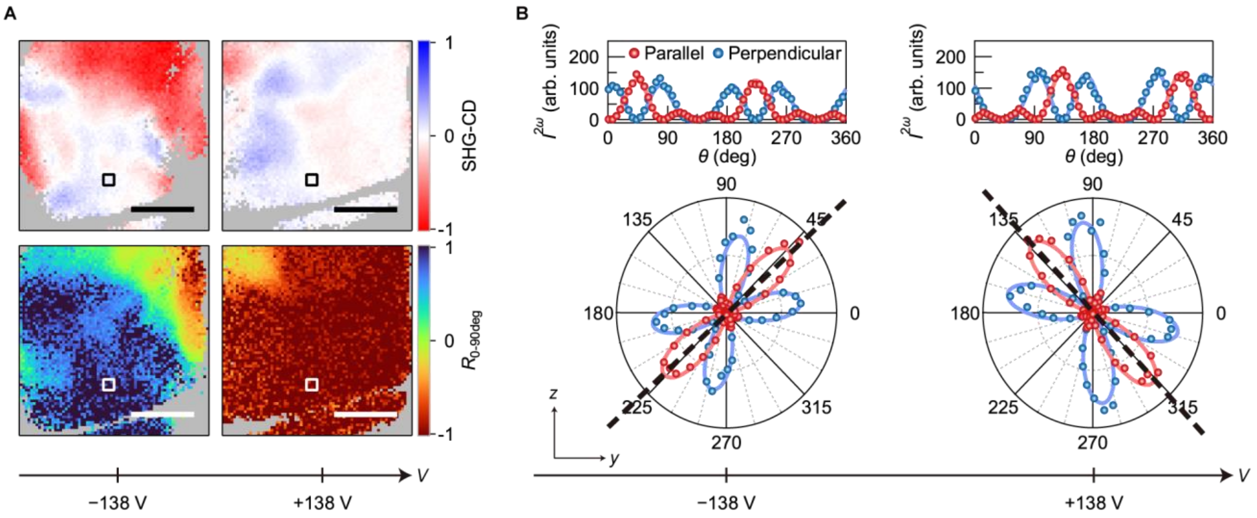

Fig. S9. Recovery of *mm*2 point group symmetry with SHG-CD ~ 0 and $\boldsymbol{R_{0-90\mathrm{deg}}}$~ ± 1. (**A**) Maps of SHG-CD (top panels) and degree of spontaneous polarization state (bottom panels) for $V = -138$ V (left panels) and +138 V (right panels). The black- (top panels) and white- (bottom panels) boxes display the same area. Scale bars, 3 μm. The data are the same as those in Figs. 4D and E in the main text. (**B**) Left panels: the top panel shows the θ dependence of the SHG intensities in the parallel (red circles) and perpendicular (blue circles) configurations of the SHG-RA measurements for $V = -138$ V. The SHG intensities were obtained by averaging the signals in the boxed region in (A). The red and blue curves are fits using Eq. 1 in the main text. The bottom panel shows the polar plots of the SHG intensities in the top panel. The black dashed line is along the crystallographic *c*-axis and shows the orientation of the glide mirror plane. Right panels: the same as the left panels but for V = +138 V.

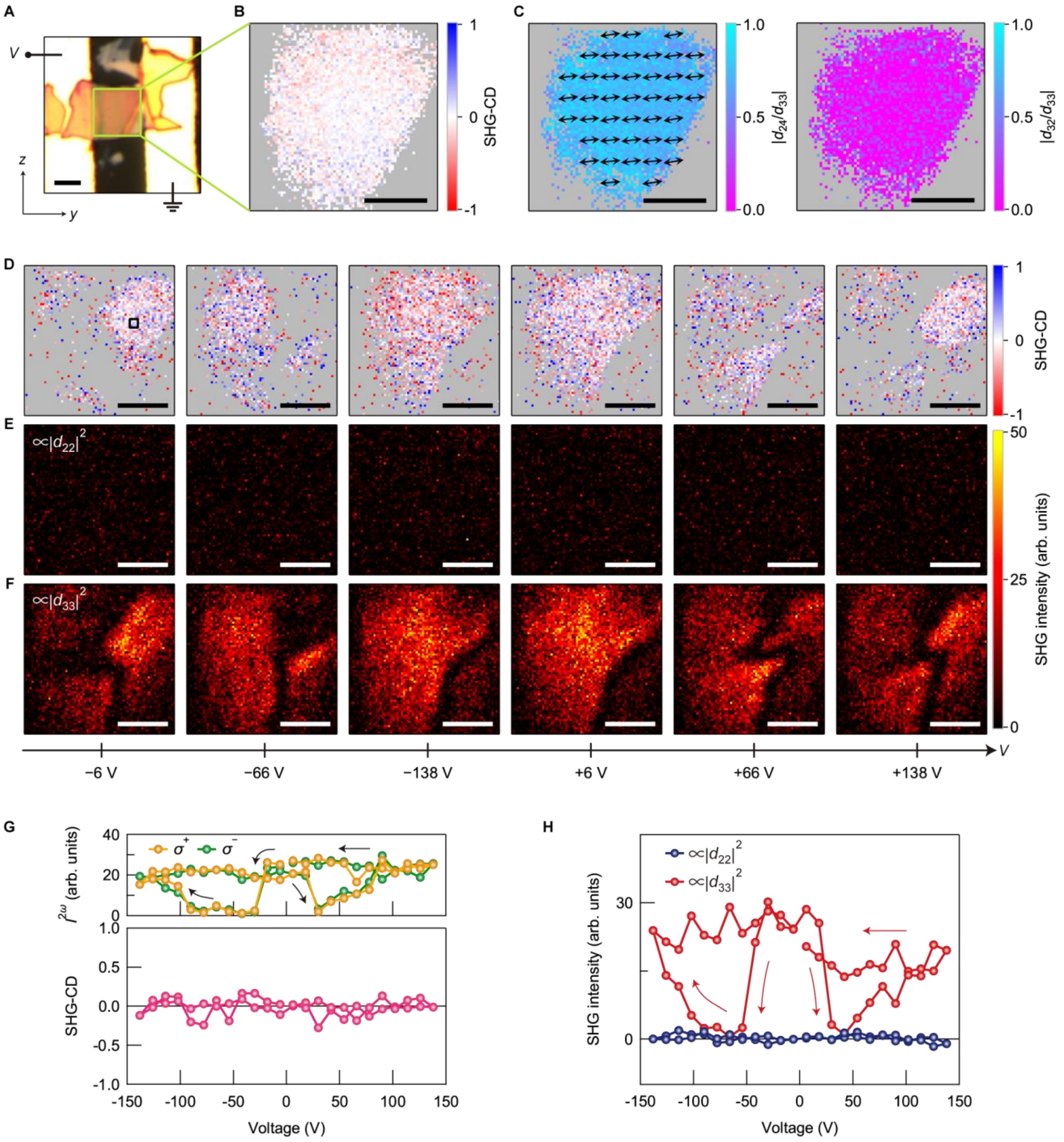

Fig. S10. Electrically induced SHG responses from $(BA)_2(EA)_2Pb_3I_{10}$ flake with its spontaneous polarization oriented perpendicular to the electrodes. (**A**) Optical microscope image of an exfoliated sample on interdigitated electrodes with the *y*- and *z*-axes of the laboratory coordinate system. The right electrode was connected to ground. Scale bar, 5 μm. (**B**) Map of the SHG-CD before applying the voltage pulses. The displayed region corresponds to the area marked by the green square in (A). Scale bar, 3 μm. (**C**) Maps of $|d_{24}|/|d_{33}|$ (left panel) and $|d_{32}|/|d_{33}|$ (right panel) before electrical poling estimated in the same spatial region as that shown in (B). The black double-sided arrows in the left panel indicate the two possible directions of the *c*-axis at each spatial position. Scale bars, 3 μm. (**D-F**) A series of maps of SHG-CD (D)

and SHG intensities proportional to $|d_{22}|^2$ (E) and $|d_{33}|^2$ (F) after applying voltage pulses with different amplitudes during the poling process. The displayed region is the same as the one shown in (B). Scale bars, 3 μm. (**G**) Hysteresis of total SHG intensities with σ^+ (top panel; orange circles) and σ^- (top panel; green circles) circularly polarized FWs and the corresponding SHG-CD (bottom panel; pink circles). The arrows indicate the switching sequence. (**H**) Hysteresis of the SHG intensities proportional to $|d_{22}|^2$ (blue circles) and $|d_{33}|^2$ (red circles). The arrows indicate the switching sequence. The SHG signals shown in (G and H) were obtained by averaging the signals in the area marked by the black square in the leftmost panel of (D). The grey-shaded areas in (B-D) correspond to the regions where the related SHG intensities are weak or not observable.

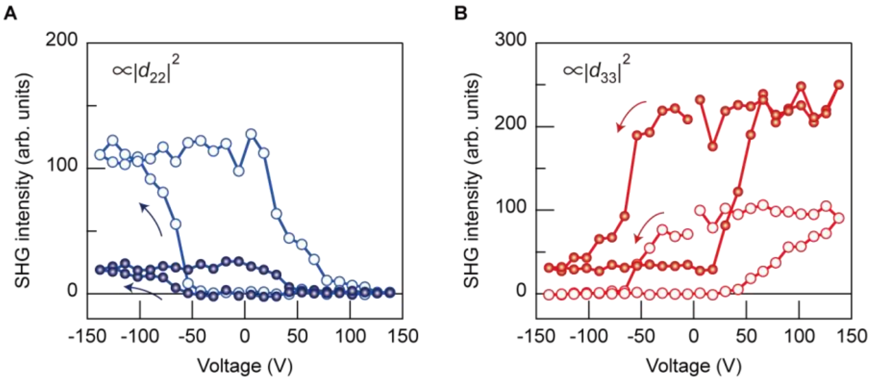

Fig. S11. Spatially dependent SHG hysteresis loops. (**A**) Hysteresis of SHG intensities proportional to $|d_{22}|^2$ obtained by averaging the signals in the area marked by the green (open circles) and purple (solid circles) squares in the leftmost panel of Fig. 4D in the main text. (**B**) Same as (A) but for SHG intensities proportional to $|d_{33}|^2$. The SHG hysteresis loops obtained from the region enclosed by the purple square (solid circles) are shifted in the negative voltage direction compared to those obtained from the region enclosed by the green square (open circles). The arrows in the panels indicate the switching sequence.

REFERENCES AND NOTES

1. K. F. Mak, J. Shan, Photonics and optoelectronics of 2D semiconductor transition metal dichalcogenides. *Nat. Photonics* **10**, 216–226 (2016).

2. Y. Liu, Y. Huang, X. Duan, Van der Waals integration before and beyond two-dimensional materials. *Nature* **567**, 323–333 (2019).

3. K. Yao, N. R. Finney, J. Zhang, S. L. Moore, L. Xian, N. Tancogne-Dejean, F. Liu, J. Ardelean, X. Xu, D. Halbertal, K. Watanabe, T. Taniguchi, H. Ochoa, A. Asenjo-Garcia, X. Zhu, D. N. Basov, A. Rubio, C. R. Dean, J. Hone, P. J. Schuck, Enhanced tunable second harmonic generation from twistable interfaces and vertical superlattices in boron nitride homostructures. *Sci. Adv.* **7**, eabe8691 (2021).

4. X. Xu, C. Trovatello, F. Mooshammer, Y. Shao, S. Zhang, K. Yao, D. N. Basov, G. Cerullo, P. J. Schuck, Towards compact phase-matched and waveguided nonlinear optics in atomically layered semiconductors. *Nat. Photonics* **16**, 698–706 (2022).

5. S. Klimmer, O. Ghaebi, Z. Gan, A. George, A. Turchanin, G. Cerullo, G. Soavi, All-optical polarization and amplitude modulation of second-harmonic generation in atomically thin semiconductors. *Nat. Photonics* **15**, 837–842 (2021).

6. C. Trovatello, A. Marini, X. Xu, C. Lee, F. Liu, N. Curreli, C. Manzoni, S. Dal Conte, K. Yao, A. Ciattoni, J. Hone, X. Zhu, P. J. Schuck, G. Cerullo, Optical parametric amplification by monolayer transition metal dichalcogenides. *Nat. Photonics* **15**, 6–10 (2021).

7. Y. Dong, M.-M. Yang, M. Yoshii, S. Matsuoka, S. Kitamura, T. Hasegawa, N. Ogawa, T. Morimoto, T. Ideue, Y. Iwasa, Giant bulk piezophotovoltaic effect in 3R-MoS_2. *Nat. Nanotechnol.* **18**, 36–41 (2023).

8. C. Wang, L. You, D. Cobden, J. Wang, Towards two-dimensional van der Waals ferroelectrics. *Nat. Mater.* **22**, 542–552 (2023).

9. I. Abdelwahab, B. Tilmann, Y. Wu, D. Giovanni, I. Verzhbitskiy, M. Zhu, R. Berté, F. Xuan, L. D. S. Menezes, G. Eda, T. C. Sum, S. Y. Quek, S. A. Maier, K. P. Loh, Giant second-harmonic generation in ferroelectric $NbOI_2$. *Nat. Photonics* **16**, 644–650 (2022).

10. Q. Guo, X.-Z. Qi, L. Zhang, M. Gao, S. Hu, W. Zhou, W. Zang, X. Zhao, J. Wang, B. Yan, M. Xu, Y.-K. Wu, G. Eda, Z. Xiao, S. A. Yang, H. Gou, Y. P. Feng, G.-C. Guo, W. Zhou, X.-F. Ren, C.-W. Qiu, S. J. Pennycook, A. T. S. Wee, Ultrathin quantum light source with van der Waals $NbOCl_2$ crystal. *Nature* **613**, 53–59 (2023).

11. A. Fieramosca, L. Polimeno, V. Ardizzone, L. De Marco, M. Pugliese, V. Maiorano, M. De Giorgi, L. Dominici, G. Gigli, D. Gerace, D. Ballarini, D. Sanvitto, Two-dimensional hybrid perovskites sustaining strong polariton interactions at room temperature. *Sci. Adv.* **5**, eaav9967 (2019).

12. C. Qin, A. S. D. Sandanayaka, C. Zhao, T. Matsushima, D. Zhang, T. Fujihara, C. Adachi, Stable room-temperature continuous-wave lasing in quasi-2D perovskite films. *Nature* **585**, 53–57 (2020).

13. H. Tsai, W. Nie, J.-C. Blancon, C. C. Stoumpos, R. Asadpour, B. Harutyunyan, A. J. Neukirch, R. Verduzco, J. J. Crochet, S. Tretiak, L. Pedesseau, J. Even, M. A. Alam, G. Gupta, J. Lou, P. M. Ajayan, M. J. Bedzyk, M. G. Kanatzidis, A. D. Mohite, High-efficiency two-dimensional Ruddlesden–Popper perovskite solar cells. *Nature* **536**, 312–316 (2016).

14. C. Qin, T. Matsushima, W. J. Potscavage, A. S. D. Sandanayaka, M. R. Leyden, F. Bencheikh, K. Goushi, F. Mathevet, B. Heinrich, G. Yumoto, Y. Kanemitsu, C. Adachi, Triplet management for efficient perovskite light-emitting diodes. *Nat. Photonics* **14**, 70–75 (2020).

15. D. Giovanni, W. K. Chong, H. A. Dewi, K. Thirumal, I. Neogi, R. Ramesh, S. Mhaisalkar, N. Mathews, T. C. Sum, Tunable room-temperature spin-selective optical Stark effect in solution-processed layered halide perovskites. *Sci. Adv.* **2**, e1600477 (2016).

16. G. Yumoto, F. Sekiguchi, R. Hashimoto, T. Nakamura, A. Wakamiya, Y. Kanemitsu, Rapidly expanding spin-polarized exciton halo in a two-dimensional halide perovskite at room temperature. *Sci. Adv.* **8**, eabp8135 (2022).

17. C. C. Stoumpos, D. H. Cao, D. J. Clark, J. Young, J. M. Rondinelli, J. I. Jang, J. T. Hupp, M. G. Kanatzidis, Ruddlesden–Popper hybrid lead iodide perovskite 2D homologous semiconductors. *Chem. Mater.* **28**, 2852–2867 (2016),.

18. J.-C. Blancon, J. Even, C. C. Stoumpos, M. G. Kanatzidis, A. D. Mohite, Semiconductor physics of organic–inorganic 2D halide perovskites. *Nat. Nanotechnol.* **15**, 969–985 (2020).

19. Y. Fu, Stabilization of metastable halide perovskite lattices in the 2D limit. *Adv. Mater.* **34**, e2108556 (2022).

20. X. Li, J. M. Hoffman, M. G. Kanatzidis, The 2D halide perovskite rulebook: How the spacer influences everything from the structure to optoelectronic device efficiency. *Chem. Rev.* **121**, 2230–2291 (2021).

21. W.-Q. Liao, Y. Zhang, C.-L. Hu, J.-G. Mao, H.-Y. Ye, P.-F. Li, S. D. Huang, R.-G. Xiong, A lead-halide perovskite molecular ferroelectric semiconductor. *Nat. Commun.* **6**, 7338 (2015).

22. Z. Sun, X. Liu, T. Khan, C. Ji, M. A. Asghar, S. Zhao, L. Li, M. Hong, J. Luo, A photoferroelectric perovskite-type organometallic halide with exceptional anisotropy of bulk photovoltaic effects. *Angew. Chem. Int. Ed.* **55**, 6545–6550 (2016).

23. P.-P. Shi, S.-Q. Lu, X.-J. Song, X.-G. Chen, W.-Q. Liao, P.-F. Li, Y.-Y. Tang, R.-G. Xiong, Two-dimensional organic–inorganic perovskite ferroelectric semiconductors with fluorinated aromatic spacers. *J. Am. Chem. Soc.* **141**, 18334–18340 (2019).

24. C.-K. Yang, W.-N. Chen, Y.-T. Ding, J. Wang, Y. Rao, W.-Q. Liao, Y.-Y. Tang, P.-F. Li, Z.-X. Wang, R.-G. Xiong, The first 2D homochiral lead iodide perovskite ferroelectrics: [*R*- and *S*-1-(4-chlorophenyl)ethylammonium]$_2$PbI$_4$. *Adv. Mater.* **31**, e1808088 (2019).

25. S. Han, X. Liu, Y. Liu, Z. Xu, Y. Li, M. Hong, J. Luo, Z. Sun, High-temperature antiferroelectric of lead iodide hybrid perovskites. *J. Am. Chem. Soc.* **141**, 12470–12474 (2019).

26. S. Wang, X. Liu, L. Li, C. Ji, Z. Sun, Z. Wu, M. Hong, J. Luo, An unprecedented biaxial trilayered hybrid perovskite ferroelectric with directionally tunable photovoltaic effects. *J. Am. Chem. Soc.* **141**, 7693–7697 (2019).

27. S. Wang, L. Li, W. Weng, C. Ji, X. Liu, Z. Sun, W. Lin, M. Hong, J. Luo, Trilayered lead chloride perovskite ferroelectric affording self-powered visible-blind ultraviolet photodetection with large zero-bias photocurrent. *J. Am. Chem. Soc.* **142**, 55–59 (2020).

28. S. Han, M. Li, Y. Liu, W. Guo, M.-C. Hong, Z. Sun, J. Luo, Tailoring of a visible-light-absorbing biaxial ferroelectric towards broadband self-driven photodetection. *Nat. Commun.* **12**, 284 (2021).

29. Y. Ma, J. Wang, W. Guo, S. Han, J. Xu, Y. Liu, L. Lu, Z. Xie, J. Luo, Z. Sun, The first improper ferroelectric of 2D multilayered hybrid perovskite enabling strong tunable polarization-directed second harmonic generation effect. *Adv. Funct. Mater.* **31**, 2103012 (2021).

30. S. Han, Y. Ma, L. Hua, L. Tang, B. Wang, Z. Sun, J. Luo, Soft multiaxial molecular ferroelectric thin films with self-powered broadband photodetection. *J. Am. Chem. Soc.* **144**, 20315–20322 (2022).

31. Y.-Y. Tang, P.-F. Li, W.-Q. Liao, P.-P. Shi, Y.-M. You, R.-G. Xiong, Multiaxial molecular ferroelectric thin films bring light to practical applications. *J. Am. Chem. Soc.* **140**, 8051–8059 (2018).

32. J. D. Byers, H. I. Yee, T. Petralli-Mallow, J. M. Hicks, Second-harmonic generation circular-dichroism spectroscopy from chiral monolayers. *Phys. Rev. B* **49**, 14643–14647 (1994).

33. C. Yuan, X. Li, S. Semin, Y. Feng, T. Rasing, J. Xu, Chiral lead halide perovskite nanowires for second-order nonlinear optics. *Nano Lett.* **18**, 5411–5417 (2018).

34. P. Behera, M. A. May, F. Gómez-Ortiz, S. Susarla, S. Das, C. T. Nelson, L. Caretta, S.-L. Hsu, M. R. McCarter, B. H. Savitzky, E. S. Barnard, A. Raja, Z. Hong, P. García-Fernandez, S. W. Lovesey, G. van der Laan, P. Ercius, C. Ophus, L. W. Martin, J. Junquera, M. B. Raschke, R. Ramesh, Electric field control of chirality. *Sci. Adv.* **8**, eabj8030 (2022).

35. Z. Guo, J. Li, J. Liang, C. Wang, X. Zhu, T. He, Regulating optical activity and anisotropic second-harmonic generation in zero-dimensional hybrid copper halides. *Nano Lett.* **22**, 846–852 (2022).

36. Z. Guo, J. Li, R. Liu, Y. Yang, C. Wang, X. Zhu, T. He, Spatially correlated chirality in chiral two-dimensional perovskites revealed by second-harmonic-generation circular dichroism microscopy. *Nano Lett.* **23**, 7434–7441 (2023).

37. P.-J. Huang, K. Taniguchi, M. Shigefuji, T. Kobayashi, M. Matsubara, T. Sasagawa, H. Sato, H. Miyasaka, Chirality-dependent circular photogalvanic effect in enantiomorphic 2D organic–inorganic hybrid perovskites. *Adv. Mater.* **33**, e2008611 (2021).

38. C. Niu, S. Huang, N. Ghosh, P. Tan, M. Wang, W. Wu, X. Xu, P. D. Ye, Tunable circular photogalvanic and photovoltaic effect in 2D tellurium with different chirality. *Nano Lett.* **23**, 3599–3606 (2023).

39. W.-F. Deng, Y.-X. Li, Y.-X. Zhao, J.-S. Hu, Z.-S. Yao, J. Tao, Inversion of molecular chirality associated with ferroelectric switching in a high-temperature two-dimensional perovskite ferroelectric. *J. Am. Chem. Soc.* **145**, 5545–5552 (2023).

40. G. Long, R. Sabatini, M. I. Saidaminov, G. Lakhwani, A. Rasmita, X. Liu, E. H. Sargent, W. Gao, Chiral-perovskite optoelectronics. *Nat. Rev. Mater.* **5**, 423–439 (2020).

41. P. Siwach, P. Sikarwar, J. S. Halpati, A. K. Chandiran, Design of above-room-temperature ferroelectric two-dimensional layered halide perovskites. *J. Mater. Chem. A* **10**, 8719–8738 (2022).

42. L. Tang, S. Han, Y. Ma, Y. Liu, L. Hua, H. Xu, W. Guo, B. Wang, Z. Sun, J. Luo, Giant near-room-temperature pyroelectric figures-of-merit originating from unusual dielectric bistability of two-dimensional perovskite ferroelectric crystals. *Chem. Mater.* **34**, 8898–8904 (2022).

43. C. Higashimura, G. Yumoto, T. Yamada, T. Nakamura, F. Harata, H. Hirori, A. Wakamiya, Y. Kanemitsu, Spontaneous polarization induced optical responses in a two-dimensional ferroelectric halide perovskite. *J. Phys. Chem. Lett.* **14**, 8360–8366 (2023).

44. Y. Fu, X. Jiang, X. Li, B. Traore, I. Spanopoulos, C. Katan, J. Even, M. G. Kanatzidis, E. Harel, Cation engineering in two-dimensional Ruddlesden–Popper lead iodide perovskites with mixed large A-site cations in the cages. *J. Am. Chem. Soc.* **142**, 4008–4021 (2020).

45. K. Koshelev, P. Tonkaev, Y. Kivshar, Nonlinear chiral metaphotonics: A perspective. *Adv. Photonics* **5**, 064001 (2023).

46. S. A. Denev, T. T. A. Lummen, E. Barnes, A. Kumar, V. Gopalan, Probing ferroelectrics using optical second harmonic generation. *J. Am. Ceram. Soc.* **94**, 2699–2727 (2011).

47. R. W. Boyd, *Nonlinear Optics* (Academic Press, ed. 3, 2008).

48. X. Fu, Z. Zeng, S. Jiao, X. Wang, J. Wang, Y. Jiang, W. Zheng, D. Zhang, Z. Tian, Q. Li, A. Pan, Highly anisotropic second-order nonlinear optical effects in the chiral lead-free perovskite spiral microplates. *Nano Lett.* **23**, 606–613 (2023).

49. S. Chen, F. Zeuner, M. Weismann, B. Reineke, G. Li, V. K. Valev, K. W. Cheah, N. C. Panoiu, T. Zentgraf, S. Zhang, Giant nonlinear optical activity of achiral origin in planar metasurfaces with quadratic and cubic nonlinearities. *Adv. Mater.* **28**, 2992–2999 (2016).

50. T. Schenk, E. Yurchuk, S. Mueller, U. Schroeder, S. Starschich, U. Böttger, T. Mikolajick, About the deformation of ferroelectric hystereses. *Appl. Phys. Rev.* **1**, 041103 (2014).

51. T. Narushima, H. Okamoto, Circular dichroism microscopy free from commingling linear dichroism via discretely modulated circular polarization. *Sci. Rep.* **6**, 35731 (2016).

52. V. Gopalan, R. Raj, Domain structure and phase transitions in epitaxial $KNbO_3$ thin films studied by in situ second harmonic generation measurements. *Appl. Phys. Lett.* **68**, 1323–1325 (1996).

53. J. Geler-Kremer, F. Eltes, P. Stark, D. Stark, D. Caimi, H. Siegwart, B. Jan Offrein, J. Fompeyrine, S. Abel, A ferroelectric multilevel non-volatile photonic phase shifter. *Nat. Photonics* **16**, 491–497 (2022).

54. S. Deng, E. Shi, L. Yuan, L. Jin, L. Dou, L. Huang, Long-range exciton transport and slow annihilation in two-dimensional hybrid perovskites. *Nat. Commun.* **11**, 664 (2020).

55. S. B. Anantharaman, C. E. Stevens, J. Lynch, B. Song, J. Hou, H. Zhang, K. Jo, P. Kumar, J.-C. Blancon, A. D. Mohite, J. R. Hendrickson, D. Jariwala, Self-hybridized polaritonic emission from layered perovskites. *Nano Lett.* **21**, 6245–6252 (2021).
